# PXR Activation Relieves Deoxynivalenol‐Induced Liver Oxidative Stress Via Malat1 LncRNA m^6^A Demethylation

**DOI:** 10.1002/advs.202308742

**Published:** 2024-04-24

**Authors:** Yue Feng, Jiakun Shen, Zishen Lin, Zeyi Chen, Min Zhou, Xi Ma

**Affiliations:** ^1^ State Key Laboratory of Animal Nutrition, College of Animal Science and Technology China Agricultural University Beijing 100193 China; ^2^ College of Life Sciences Henan Agricultural University Zhengzhou 450046 China

**Keywords:** deoxynivalenol, indole‐3‐propionic acid, Malat1 lncRNA, N6‐methyladenosine, pregnane X receptor

## Abstract

Deoxynivalenol (DON) is a prevalent toxin causing severe liver damage through hepatocellular oxidative stress. However, the underlying mechanisms and effective therapeutic approaches remain unknown. Here, the unique role of the xenobiotic metabolism factor pregnane X receptor (PXR) in mediating DON‐induced hepatocellular oxidative stress is investigated. Treatment with the PXR agonist 3‐indole‐propionic acid (IPA) alleviates DON‐induced oxidative stress and liver injury both in vitro and in vivo. Mechanistically, it is discovered for the first time that PXR agonist IPA directly transactivates the m^6^A demethylase FTO expression, leading to site‐specific demethylation and decreased abundance of YTHDC1‐bound Malat1 lncRNA at single‐nucleotide resolution. The diminished m^6^A modification of Malat1 lncRNA reduces its stability and augments antioxidant pathways governed by NRF2, consequently mitigating DON‐induced liver injury. Furthermore, Malat1 knockout mice exhibit decreased DON‐induced liver injury, emphasizing the role of Malat1 lncRNA in oxidative stress. Collectively, the findings establish that PXR‐mediated m^6^A‐dependent Malat1 lncRNA expression determines hepatocyte oxidative stress via m^6^A demethylase FTO, providing valuable insights into the potential mechanisms underlying DON‐induced liver injury and offers potential therapeutic strategies for its treatment.

## Graphical abstract

To explore the mechanism of PXR in hepatocyte detoxification, cells are treated with deoxynivalenol, and PXR agonist IPA is administered. IPA activates FTO, leading to the m^6^A demethylation of Malat1 lncRNA. The reduced stability of Malat1 enhances NRF2‐mediated antioxidant function, thereby mitigating deoxynivalenol‐induced liver damage. This uncovers the PXR‐FTO‐Malat1‐NRF2 pathway, highlighting IPA as a potential agent for liver detoxification.

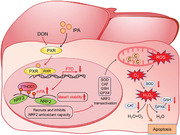

## 1 Introduction

Deoxynivalenol (DON) is a prevalent mycotoxin contaminant found in human food and animal feed, primarily produced by field pathogens such as *F. culmorum* and *F. graminearum*.^[^ ^1^ ^]^ Global studies have revealed that DON contamination affects ≈64% of grain samples, with alarming rates as high as 96.4% in feed samples from China,^[^ ^2^ ^]^ posing significant threats to food safety for both animals and humans. The toxicity of DON involves oxidative stress‐mediated DNA damage and apoptosis, as well as the inhibition of protein synthesis due to the binding of peptidyl‐transferase.^[^ ^3^ ^]^ While the small intestine serves as the initial site of DON exposure and absorption, it does not significantly contribute to DON accumulation and detoxification.^[^ ^4^ ^]^ Conversely, upon absorption, DON is efficiently transported to the liver, which serves as the primary organ for DON accumulation and detoxification.^[^ ^5^ ^]^ Consequently, excessive exposure to DON can result in liver damage and hepatitis.^[^ ^6^ ^]^ Given the critical role of the liver in DON metabolism, there is an urgent need to explore novel strategies to mitigate DON toxicity and improve resistance to DON‐induced liver injury.

The metabolism of ingested DON involves primary glucuronidation, mediated by pregnane X receptor (PXR)‐regulated UDP‐glucuronosyltransferases (UGTs) within hepatocytes, followed by urinary excretion.^[^ ^7^ ^]^ PXR, a member of the nuclear receptor protein family, plays a crucial role in modulating various metabolic processes, including drug clearance, inflammation, cell differentiation, and oxidative stress, either through direct binding to genomic regions or indirect crosstalk with other transcriptional factors.^[^ ^8^ ^]^ Given the involvement of PXR in recognizing and eliminating endogenous and exogenous substances, prolonged exposure to DON may disrupt normal PXR expression and function, leading to excessive oxidative stress and damage in hepatocytes. Therefore, targeted drugs that modulate PXR expression and activity could serve as potential therapeutic approaches for treating DON‐induced liver injury. However, research on the expression patterns of PXR in DON‐induced liver damage, as well as its potential mechanisms in regulating hepatocellular oxidative stress, remains limited and requires further investigation.

To investigate the regulatory role of PXR in DON‐induced hepatic oxidative stress, we conducted a transcriptomic analysis to examine differential gene expression in hepatocytes from control, PXR agonist‐treated, and the DON‐treated groups. Notably, the expression of Metastasis‐associated lung adenocarcinoma transcript 1 (Malat1) lncRNA was significantly altered in the DON and PXR agonist treatment groups compared to the control, implying that Malat1 could be a target gene of PXR in regulating DON‐induced hepatic oxidative stress. Long non‐coding RNAs (lncRNAs) have been shown to play crucial roles as biomarkers and transcriptional regulators in oxidative stress and inflammatory responses.^[^ ^9^ ^]^ In particular, Malat1 lncRNA has been implicated in antioxidant reactions, exhibiting both positive and negative regulatory effects in different cellular contexts. Previous research has demonstrated that Malat1 lncRNA can reduce oxidative stress in human umbilical vein endothelial cells (HUVECs) by inhibiting Keap1 activation and stabilizing NRF2 protein expression.^[^ ^10^ ^]^ However, in hepatocytes, Malat1 has been shown to act as a negative regulator of NRF2 transcriptional activity. Malat1 lncRNA preferentially binds to NRF2, leading to its inactivation and increased oxidative stress.^[^ ^11^ ^]^ Knockout of Malat1 suggests that targeting the NRF2‐Keap1‐ARE pathway could be a critical strategy for regulating oxidative stress.^[^ ^11^ ^]^ We speculate that PXR may participate in the process of DON‐induced hepatic oxidative stress and liver injury by regulating the expression of Malat1 lncRNA. However, the specific molecular mechanisms by which PXR regulates Malat1 lncRNA have yet to be elucidated.

Epigenetic modifications, including histone modification, DNA methylation, and microRNA regulation, have been implicated in various human diseases and animal toxicities induced by DON mycotoxin.^[^ ^12^ ^]^ However, the relationship between RNA methylation and DON‐induced liver toxicity remains unclear. N6‐methyladenosine (m^6^A) is the most prevalent chemical modification in the transcriptome and dynamically regulates RNA translation, splicing, and stability.^[^ ^13^ ^]^ Recent studies have linked m^6^A modifications to liver diseases, such as fatty liver,^[^ ^14^ ^]^ hepatitis,^[^ ^15^ ^]^ cirrhosis,^[^ ^16^ ^]^ and HCC.^[^ ^17^ ^]^ Given the known involvement of m^6^A in liver diseases and its association with oxidative stress,^[^ ^18^ ^]^ it is plausible that m^6^A modification may also play a role in DON‐induced hepatic oxidative stress and liver injury. Notably, the highly conserved region of Malat1 lncRNA contains clusters of m^6^A modifications.^[^ ^19^ ^]^ These modifications have been shown to impact the stability and function of Malat1 lncRNA in various cellular processes, including cancer cell proliferation and migration.^[^ ^20^ ^]^ Hence, exploring the potential regulatory effects of Malat1‐m^6^A modification in DON‐induced hepatic oxidative stress holds significant scientific value.

In this study, we utilized hepatic cell lines, primary mouse hepatocytes, and animal models to elucidate the critical role of the PXR‐m^6^A‐Malat1‐NRF2 signaling pathway in preventing and attenuating DON‐induced hepatic oxidative stress and injury. Furthermore, we explored the potential therapeutic effects of IPA, a PXR agonist produced by gut microbiota, as a potential new strategy for the treatment of DON‐induced hepatic injury and oxidative stress.

## 2 Results

### 2.1 PXR and Downstream Transcripts Decreased in DON‐Induced Hepatocyte Oxidative Stress

**Figure 1:**
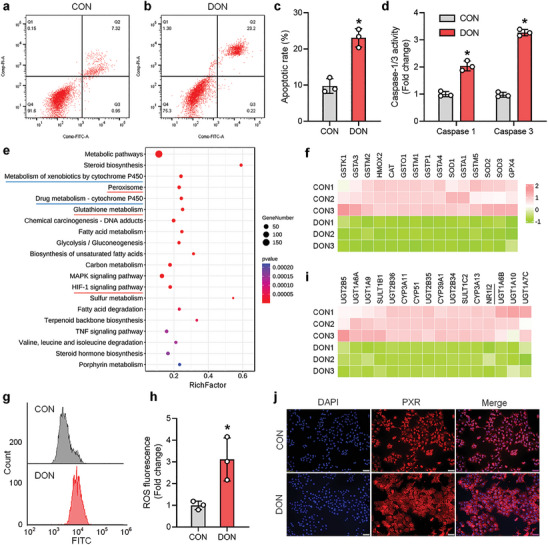
Decreased expression levels of PXR and its downstream transcripts during DON‐induced oxidative stress in hepatocytes. a–c) AML12 cells were treated with 10 nmol mL^−1^ DON for 12 h and apoptosis rates were measured by flow cytometry (n = 3). d) The activity of apoptotic proteases caspase‐1 and caspase‐3 was measured (n = 3). e) RNA‐seq analysis of DON‐treated AML12 cells showed significant enrichment of genes related to oxidative stress pathways, such as peroxisome and glutathione metabolism, as well as detoxification pathways mediated by PXR, such as metabolism of xenobiotics by cytochrome P450 and drug metabolism‐cytochrome P450 (n = 3). f,i) Heatmap analysis was performed to examine transcriptional changes of anti‐oxidative factors and PXR and its downstream transcripts (n = 3). g,h) ROS levels were measured and analyzed ROS levels were measured and analyzed (n = 3). j) Immunofluorescence was used to detect the cellular localization of PXR after DON treatment; DAPI dye was used to mark the location of nuclei. Values are presented as means ± SD, *P < 0.05.

Following DON treatment, AML12 cells exhibited significant apoptosis (**Figure** **1**a–c) and increased activity of apoptosis‐related factors Caspase1 and Caspase3 (Figure 1d). To investigate the molecular pathway underlying DON‐induced hepatocyte apoptosis, we conducted RNA‐seq on cell samples from the control and DON‐treated groups. KEGG analysis revealed significant enrichment of oxidative stress‐related pathways in the DON‐treated group, including Peroxisome, Glutathione metabolism, and HIF‐1 signaling pathway (Figure 1e, highlighted in red). This enrichment was particularly evident in antioxidant‐related factors such as SOD1, CAT, GPX4, and other transcripts, which displayed significantly decreased expression levels (Figure 1f). Furthermore, ROS staining with CellROX Green Reagent demonstrated that DON significantly elevated cellular ROS levels (Figure 1g,h), indicating impaired antioxidant capacity in hepatocytes, exacerbated oxidative stress, and subsequent cell apoptosis. Additionally, RNA‐seq analysis revealed notable changes in the PXR‐mediated CYP450 pathway, specifically in Metabolism of xenobiotics by cytochrome P450 and Drug metabolism‐cytochrome P450 (Figure 1e, highlighted in blue). PXR (NR1I2) and its downstream transcripts, including CYP3A and UGT1A (Figure 1i), exhibited a significant reduction following DON treatment. Moreover, cell immunofluorescence results demonstrated that DON treatment hindered the nuclear entry of PXR protein, thereby inhibiting its transcriptional activity (Figure 1j).

Similar observations were observed in primary hepatocytes treated with DON, which induced significant apoptosis (Figure S1a,b, Supporting Information), increased cellular ROS levels (Figure S1c,d, Supporting Information), and downregulated the expression levels of antioxidant‐related transcripts, including GSTO1, GSTA1, HMOX2, GPX4, SOD1, and CAT (Figure S1e–j, Supporting Information). Concurrently, the mRNA levels of PXR and its downstream factors, including CYP3A and UGT1A1, were significantly reduced (Figure S1k–n, Supporting Information). In summary, these results suggest that PXR expression and activity were diminished during the DON‐induced oxidative stress and apoptosis process in hepatocytes. Considering that PXR recognizes endogenous and exogenous compounds, modulating drug metabolism and detoxification, it is reasonable to speculate that PXR may play a regulatory role in the DON‐induced oxidative stress process in hepatocytes.

### 2.2 PXR Determines DON‐Induced Oxidative Stress in Hepatocytes

We proceeded to investigate the expression patterns of PXR in various hepatocyte oxidative stress models. Our results revealed a significant upregulation of PXR expression levels in classic hydrogen peroxide (H_2_O_2_)‐induced oxidative stress models in both primary hepatocytes (Figure S2a–e, Supporting Information) and AML12 cells (Figure S2i–m, Supporting Information). Correspondingly, PXR‐mediated transactivation of genes associated with drug metabolism and detoxification pathways was also markedly increased (Figure S2f–h and n–p, Supporting Information). In contrast, the expression pattern of PXR and its downstream transcripts in H_2_O_2_ and DON‐induced oxidative stress models in hepatocytes exhibited a complete opposite trend, suggesting a potential variation in the role of PXR across different types of hepatocyte oxidative stress models. Specifically, we hypothesized that PXR inhibition might regulate the DON‐induced oxidative stress process in hepatocytes.

**Figure 2:**
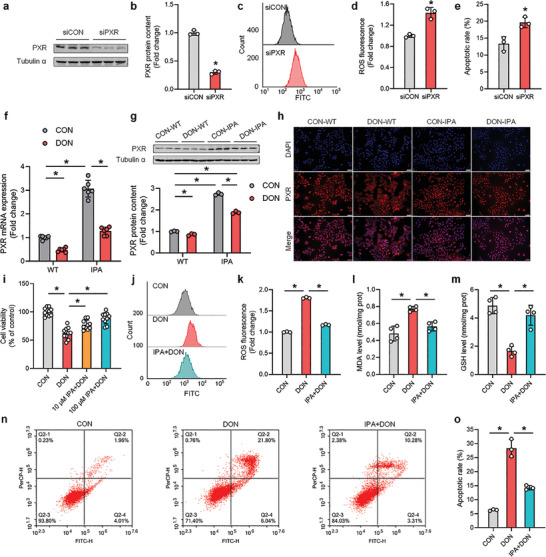
PXR regulates DON‐induced oxidative stress in hepatocytes. a,b) Validation of siRNA‐mediated PXR knockdown (n = 3). c–e) Knockdown of PXR in hepatocytes resulted in increased ROS content and apoptosis rate (n = 3). f,g) Treatment with the PXR agonist, IPA, mitigated the decrease in both mRNA (n = 6) and protein (n = 3) levels of PXR caused by DON. h) IPA treatment mitigated the inability of PXR to translocate into the nucleus after DON treatment. i) Treatment with different concentrations of IPA mitigated the decrease in hepatocyte viability caused by DON (n = 10). j–o) IPA treatment alleviated excessive ROS accumulation, disturbance in oxidative stress key enzyme MDA and GSH levels, and significant increase in apoptosis rates caused by DON (n = 3). Values are presented as means ± SD, *P < 0.05.

To investigate the regulatory role of PXR in DON‐induced oxidative stress in hepatocytes, we initially employed siRNA to knockdown PXR in AML12 cells (**Figure** **2**a,b; Figure S3b, Supporting Information). The results demonstrated that PXR knockdown directly led to a significant increase in ROS levels (Figure 2c and d) and apoptotic rate (Figure 2e). RNA‐seq analysis unveiled a significant enrichment of oxidative stress‐related pathways (Figure S3a, Supporting Information), accompanied by a significant reduction in the expression levels of antioxidant‐related genes (Figure S3c–e, Supporting Information). Conversely, treatment with the PXR agonist IPA (Figure S3f, Supporting Information) reversed the decrease in PXR expression and activity induced by DON in hepatocytes (Figure 2f–h). Furthermore, IPA dose‐dependently mitigated the decline in cell viability caused by DON (Figure 2i), suppressed the excessive generation of ROS and the key oxidative stress enzyme MDA (Figure 2j–l), enhanced the content of the antioxidant key enzyme GSH (Figure 2m), and ultimately alleviated DON‐induced hepatocyte apoptosis (Figure 2n,o). We also tested another well‐known PXR agonist, rifampicin (RIF, Figure S3g, Supporting Information), which partially alleviated the decrease in cell viability (Figure S3h, Supporting Information) and inhibited ROS content and oxidative stress (Figure S3i–l, Supporting Information). However, the effect of RIF was less pronounced compared to IPA treatment. In conclusion, these findings suggest that PXR plays a significant role in modulating DON‐induced oxidative stress in hepatocytes, and the use of the PXR agonist IPA may offer a promising approach to alleviate the adverse effects of DON‐induced oxidative stress in the liver.

### 2.3 Malat1 lncRNA Regulates DON‐Induced Hepatocyte Oxidative Stress

**Figure 3:**
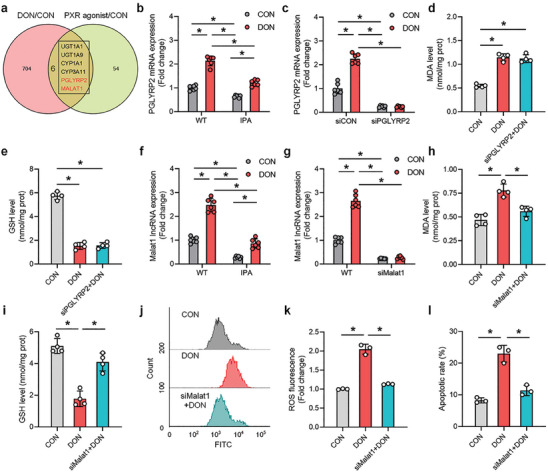
Malat1 lncRNA regulates PXR‐mediated DON‐induced hepatocyte oxidative stress. a) Intersection analysis of differentially expressed genes in RNA‐seq data from DON‐treated hepatocytes and PXR agonist PCN‐treated hepatocytes. b) Effects of IPA and DON alone or in combination on PGLYRP2 mRNA levels (n = 6). c) Validation of siRNA transfection efficiency for PGLYRP2 knockdown and its impact on PGLYRP2 mRNA levels after DON treatment (n = 6). d,e) Effects of PGLYRP2 knockdown on the levels of oxidative stress key enzymes MDA/GSH in hepatocytes treated with DON (n = 3 or 4). f) Effects of IPA and DON alone or in combination on Malat1 lncRNA expression levels (n = 6). g) Validation of siRNA transfection efficiency for Malat1 lncRNA knockdown and its impact on PGLYRP2 mRNA levels after DON treatment (n = 6). h–l) The mitigating effect of Malat1 lncRNA knockdown on oxidative stress‐related markers and apoptosis rates in hepatocytes induced by DON (n = 3 or 4). Values are presented as means ± SD, *P < 0.05.

To elucidate the specific molecular mechanism underlying PXR regulation of DON‐induced oxidative stress in hepatocytes, we conducted an intersection analysis of RNA‐seq results from cells treated with DON and RIF^[^ ^21^ ^]^ (**Figure** **3**a). The analysis revealed six genes that exhibited significant changes in expression (either increased or decreased by more than a 3‐fold change) following both DON and PXR agonist treatments, including UGT1A1/9, CYP1A1, and CYP3A11, which are downstream targets of PXR involved in phase I/II detoxification reactions.^[^ ^22^ ^]^ However, the regulatory role and mechanism of PXR in PGLYRP2 and Malat1 lncRNA remained unknown, and their contribution to DON‐induced hepatocyte oxidative stress remained unclear.

To investigate whether PGLYRP2 and Malat1 regulate DON‐induced hepatocyte oxidative stress, we examined their expression patterns. DON treatment significantly increased the mRNA expression level of PGLYRP2, which was alleviated by treatment with the PXR agonist IPA (Figure 3b). However, knockdown of PGLYRP2 (Figure 3c) did not alleviate DON‐induced hepatocyte oxidative stress, as evidenced by the inability to inhibit changes in the key oxidative stress enzymes MDA/GSH (Figure 3d and e). In contrast, Malat1 lncRNA exhibited a significant upregulation in response to DON treatment, which was significantly reversed by IPA treatment (Figure 3f). Knockdown of Malat1 expression (Figure 3g; Figure S4c, Supporting Information) significantly attenuated DON‐induced hepatocyte oxidative stress, as evidenced by the alleviation of MDA/GSH changes (Figure 3h,i), reduction in intracellular ROS levels (Figure 3j,k), and decrease in DON‐induced hepatocyte antioxidant stress‐related factors (Figure S5a–f, Supporting Information) and apoptosis (Figure 3l). Additionally, knockdown of Malat1 lncRNA directly enhanced the antioxidant stress capacity of hepatocytes. RNA‐seq results following transfection with Malat1 siRNA revealed a significant enrichment of genes related to oxidative stress pathways in hepatocytes (Figure S4a, Supporting Information), particularly those associated with the NRF2 antioxidant pathway (Figure S4b, Supporting Information). qPCR results further confirmed that Malat1 knockdown significantly increased the transcript expression levels of the key antioxidant enzymes SOD1/CAT (Figure S4d,e, Supporting Information). In summary, these results suggest that Malat1 lncRNA, as a downstream factor of PXR, regulates DON‐induced hepatocyte oxidative stress.

### 2.4 Malat1 lncRNA Regulates DON‐Induced Hepatocyte Oxidative Stress by Binding to and Inhibiting NRF2 Antioxidant Activity

**Figure 4:**
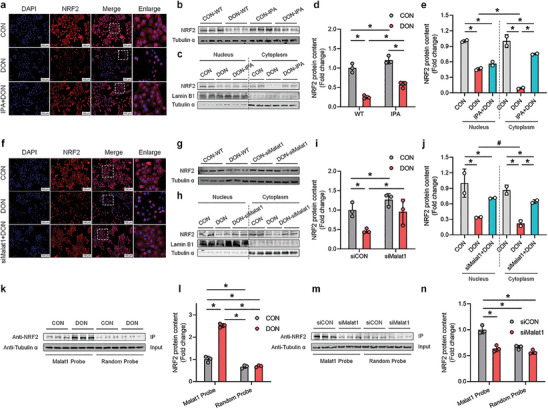
Malat1 lncRNA regulates DON‐induced hepatocyte oxidative stress by binding to and inhibiting NRF2 antioxidant activity. a) Localization of NRF2 protein in cells in the hepatocyte oxidative stress model induced by DON, mitigated by IPA. b,d) Expression levels of NRF2 protein in the hepatocyte oxidative stress model induced by DON, mitigated by IPA (n = 3). c,e) Detection of NRF2 protein expression levels in the nucleus and cytoplasm under IPA and DON treatment. f) Localization of NRF2 protein in cells under Malat1 knockdown and DON treatment. g,i) Expression levels of NRF2 protein under Malat1 knockdown and DON treatment (n = 3). h,j) Detection of NRF2 protein expression levels in the nucleus and cytoplasm under Malat1 knockdown and DON treatment. k,l) RNA affinity purification analysis of the interaction between NRF2 protein and Malat1 mRNA under DON treatment (n = 3). m, n) RNA affinity purification analysis of the interaction between NRF2 protein and Malat1 mRNA under Malat1 knockdown (n = 3). Values are expressed as mean ± standard deviation, *P < 0.05.

We investigated the involvement of Malat1 lncRNA in the regulation of DON‐induced oxidative stress in hepatocytes. RNA‐seq and qPCR assays revealed that knockdown of Malat1 led to a significant upregulation of genes related to NRF2‐mediated antioxidant pathways, indicating a potential role of Malat1 lncRNA in modulating NRF2 activity during DON‐induced oxidative stress. To further elucidate this mechanism, we examined the expression levels and activity of NRF2 in an oxidative stress model induced by DON, with and without IPA pretreatment. The results demonstrated that DON treatment significantly decreased NRF2 protein levels in hepatocytes, which was partially restored by IPA pretreatment (Figure 4b and d). Immunofluorescence analysis confirmed the reduction of NRF2 protein levels in the cytoplasm upon DON treatment, which was partially alleviated by IPA pretreatment (**Figure** **4**a). Western blot analysis further validated the decrease in cytoplasmic NRF2 protein levels under DON treatment, with partial relief observed after IPA pretreatment (Figure 4c and e).

We then investigated the effect of Malat1 knockdown on DON‐induced inhibition of NRF2 protein expression. Similar to the relief observed with IPA pretreatment, knockdown of Malat1 partially alleviated the decrease in cytoplasmic NRF2 protein levels induced by DON in hepatocytes (Figure 4f–j). Based on these results, we hypothesized that the upregulation of Malat1 lncRNA mediated by the PXR‐FTO pathway under DON treatment might lead to its binding to NRF2 protein in the nucleus, thereby preventing NRF2 from functioning as a transcription factor. To test this hypothesis, we performed Malat1 lncRNA affinity isolation to analyze the interaction between Malat1 lncRNA and NRF2 protein. The results demonstrated a significant increase in the binding abundance between Malat1 and NRF2 protein in hepatocytes after DON treatment compared to the control group (Figure 4k,l). Additionally, knockdown of Malat1 lncRNA significantly inhibited the binding abundance of Malat1 lncRNA and NRF2 protein (Figure 4m,n). Taken together, our findings suggest that DON treatment led to excessive binding of Malat1 lncRNA with the NRF2 protein and inhibiting its activity. Consequently, the antioxidant capacity of hepatocytes was significantly suppressed, resulting in increased oxidative stress.

### 2.5 PXR Regulates Malat1 lncRNA Expression Through Post‐Transcriptional m^6^A Modification in DON‐Induced Hepatocyte Oxidative Stress

**Figure 5:**
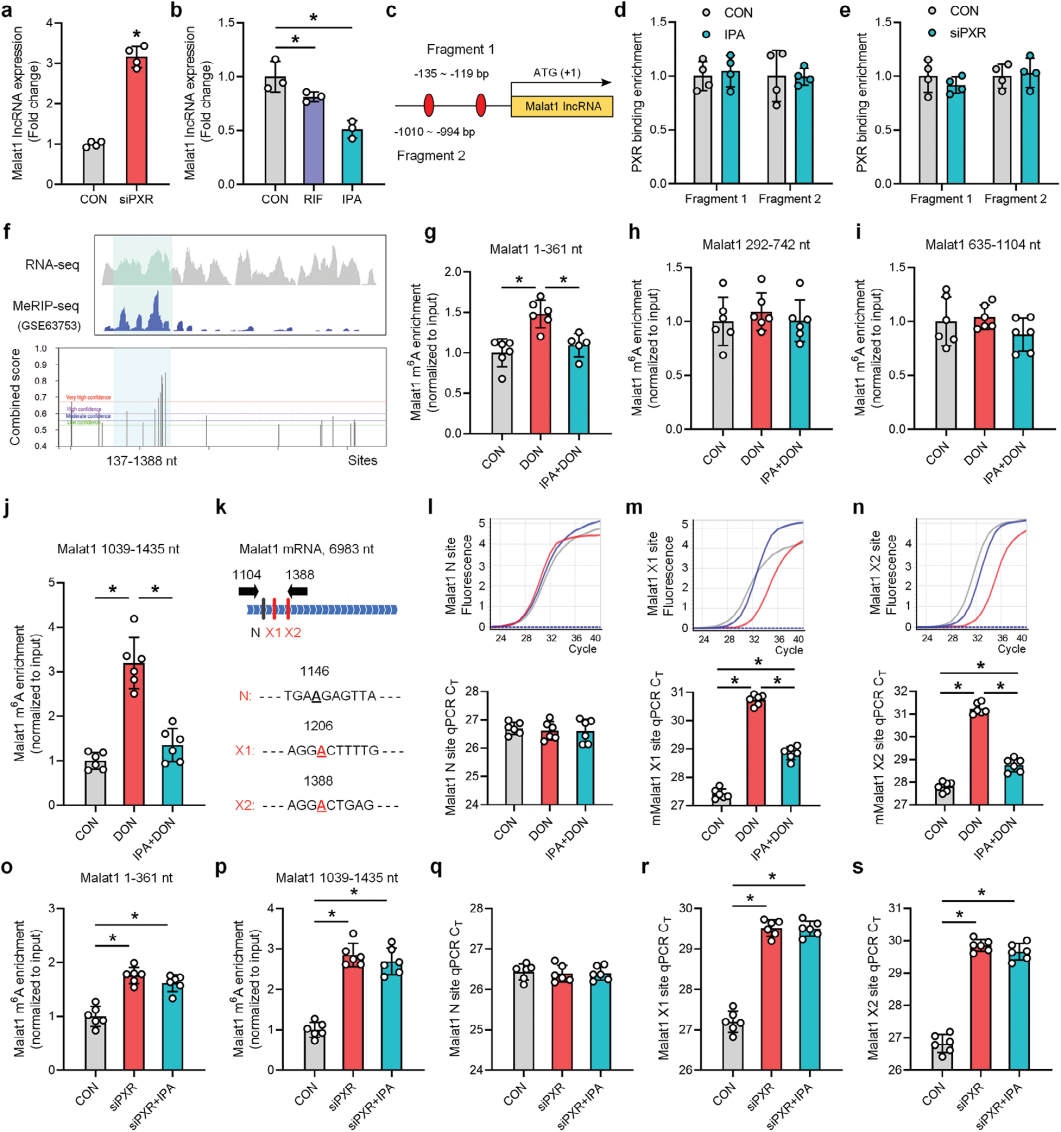
PXR regulates Malat1 lncRNA expression through m^6^A modification in DON‐induced hepatocyte oxidative stress. a) The impact of PXR knockdown on Malat1 lncRNA expression levels (n = 4). b) The impact of the PXR agonists RIF/IPA on Malat1 lncRNA expression levels (n = 3). c) Schematic diagram of potential PXR protein binding sites in the Malat1 lncRNA promoter region. Fragment 1 and 2 represent two possible binding sites. d,e) Analysis of changes in PXR protein binding abundance to potential binding sites in the Malat1 promoter region after PXR agonist treatment or knockdown using ChIP‐PCR (n = 4). f) m^6^A residues in Malat1 lncRNA were concentrated in conserved regions determined by MeRIP‐seq (GSE63753) and RNA‐seq databases (GSE63753). The m^6^A sites were predicted to be in the sequence between 137–1388 nt in SRAMP website. g–j) RNA sequences containing the 137–1388 nt fragment were divided into 4 consecutive small fragments, named Malat1 1–361 nt, Malat1 292–742 nt, Malat1 635–1104 nt, and Malat1 1039–1435 nt, and MeRIP‐qPCR was used to detect their m^6^A abundance after IPA and DON treatment (n = 6). k) SRAMP website was used to predict the specific m^6^A sites in the Malat1 1039–1435 nt fragment, and two potential m^6^A modification sites were identified and named X1 (No. 1206 nt) and X2 (No. 1388 nt), respectively. The negative control site that did not meet the m^6^A “RRACU” motif requirement was named the N site (No. 1146 nt). i–n) The SELECT method was used to detect m^6^A modifications in the Malat1 lncRNA m^6^A modification site (n = 6). o,p) MeRIP‐qPCR was used to detect their m^6^A abundance after siPXR and IPA treatment (n = 6). q–s) The SELECT method was used to detect m^6^A modifications in the Malat1 lncRNA m^6^A modification site (n = 6). Values are presented as means ± SD, *P < 0.05.

To further investigate the regulatory mechanism of PXR on Malat1 lncRNA, we performed PXR knockdown using PXR siRNA and observed a significant increase in Malat1 lncRNA expression (**Figure** **5**a). Conversely, treatment with the PXR agonist RIF/IPA led to a significant decrease in Malat1 lncRNA expression, with IPA exhibiting a stronger inhibitory effect than RIF (Figure 5b). These findings confirm that PXR negatively regulates the expression of Malat1 lncRNA.

Next, we explored the specific mechanism by which PXR regulates Malat1 lncRNA expression in the DON‐induced hepatocyte oxidative stress model. We initially hypothesized that PXR may exert its regulation through transcriptional mechanisms. However, ChIP‐PCR analysis showed no significant changes in the binding abundance of PXR protein at potential sites in the Malat1 promoter region (Figure 5c–e), indicating that the regulatory mechanism of PXR on Malat1 lncRNA expression is not transcriptional. Consequently, we examined post‐transcriptional regulatory mechanisms and identified high‐potential m^6^A peaks on the Malat1 lncRNA sequence, particularly at positions 137–1388 and 1000–1388 nt (Figure 5f). These findings suggest the presence of m^6^A modifications on Malat1 lncRNA.

To determine whether the m^6^A modification level of Malat1 lncRNA changes in the PXR‐mediated DON‐induced oxidative stress model, we artificially divided the long sequence of Malat1 lncRNA at positions 137–1388 nt into four smaller fragments and subjected them to MeRIP‐qPCR analysis. The results revealed a significant increase in the abundance of m^6^A at the 1039–1435 nt region of Malat1 lncRNA after DON treatment, which was alleviated after IPA treatment (Figure 5g–j). Utilizing the SRAMP database, we identified two highly potential m^6^A modification sites, X1 site (1206 nt) and X2 site (1388 nt), within the 1039–1435 nt region of Malat1 lncRNA (Figure 5k). SELECT analysis demonstrated that the m^6^A modification levels at the X1 and X2 sites were significantly increased after DON treatment and relieved after IPA treatment compared to the negative control (N site) (Figure 5l–n). These experiments revealed that the m^6^A modification level at specific sites of Malat1 lncRNA is significantly increased after DON treatment and regulated by the PXR agonist IPA. Furthermore, MeRIP‐qPCR results indicated that PXR knockdown significantly increased the m^6^A abundance in the 1–361 and 1039–1435 nt regions of Malat1 lncRNA (Figure 5o,p). Additionally, the SELECT assay revealed that PXR knockdown specifically enhanced the RNA methylation levels at the X1 site (1206 nt) and X2 site (1388 nt) of Malat1 lncRNA (Figure 5q–s). Interestingly, after PXR knockdown, subsequent IPA treatment on hepatocytes did not alleviate the elevated m^6^A modification levels of Malat1 lncRNA (Figure 5o–s). Overall, our results suggest that PXR regulates Malat1 lncRNA expression through post‐transcriptional regulation mechanisms, specifically through m^6^A modification at specific sites.

### 2.6 PXR Transcriptionally Activates FTO Expression, Regulating m^6^A Modification and Expression of Malat1 lncRNA in Hepatocytes

**Figure 6:**
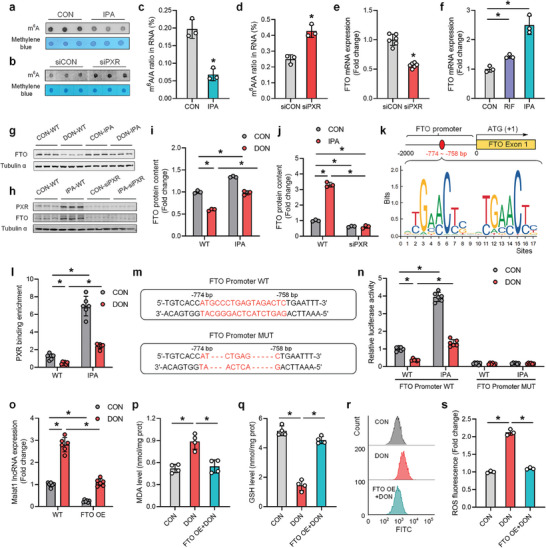
PXR transcriptionally activates FTO expression and regulates the m^6^A modification and expression level of Malat1 lncRNA in hepatocytes. a,b) The effect of PXR agonist IPA or knockdown treatment on the abundance of m^6^A modification in total RNA of AML12 cells was detected by dot blot (n = 3). c,d) The effect of PXR agonist IPA or knockdown treatment on the m^6^A/A ratio in RNA of AML12 cells was detected by LC‐MS/MS (n = 3). e,f) Changes in FTO mRNA expression levels after PXR agonist IPA or knockdown treatment (n = 3). g,i) The effect of DON and IPA alone or in combination on the expression of FTO protein (n = 3). h,j) The effect of PXR knockdown and IPA alone or in combination on the expression of FTO protein (n = 3). k) Schematic diagram of the potential PXR binding sites in the FTO gene promoter region. l) The effect of DON and IPA alone or in combination on the abundance of PXR protein binding to the FTO promoter region detected by ChIP‐PCR (n = 6). m) Schematic diagram of the dual‐luciferase activity system constructed for the FTO promoter region. Plasmids retaining the potential PXR protein binding site sequence are named WT, while plasmids with deleted potential PXR protein binding site sequences are named MUT. n) Analysis of dual‐luciferase activity after DON and IPA treatment (n = 6). o) The mitigating effect of FTO overexpression on the increase in Malat1 lncRNA expression caused by DON (n = 6). p–s) The mitigating effect of FTO overexpression on oxidative stress‐related indicators in hepatocytes caused by DON (n = 3 or 4). Values are presented as means ± SD, *P < 0.05.

To investigate the regulatory role of PXR in hepatocytes, specifically its impact on m^6^A modification and expression levels of Malat1 lncRNA, we treated AML12 cells with the PXR agonist IPA or performed PXR siRNA transfection. We collected RNA samples for m^6^A Dot blot analysis and LC‐MS/MS measurement. The results demonstrated that activating PXR significantly reduced the overall m^6^A modification level in hepatocytes, whereas PXR knockdown significantly increased the total m^6^A modification level (**Figure** **6**a–d). As the m^6^A modification level is dynamically regulated by m^6^A methyltransferases (METTL3, METTL14, and WTAP) and demethylases (FTO and ALKBH5), we further examined the effects of PXR activation or knockdown on the expression levels of these enzymes. RNA‐seq analysis indicated that PXR knockdown specifically reduced the transcript expression of the demethylase FTO, while having no effect on the mRNA levels of other m^6^A modification‐related enzymes (Figure S6a, Supporting Information). qPCR results confirmed a significant decrease in FTO expression after PXR knockdown (Figure 6e). Conversely, PXR agonists IPA and RIF significantly increased FTO mRNA levels, with IPA exhibiting a more pronounced effect (Figure 6f), and had no impact on the expression levels of METTL3/14, WATP, and ALKBH5 (Figure S6b–e, Supporting Information). In the DON‐induced hepatocyte oxidative stress model, FTO mRNA and protein expression levels were significantly reduced, and treatment with the PXR agonist IPA restored their levels (Figure 6g,i; Figure S6j, Supporting Information), while having no effect on the mRNA and protein levels of other m^6^A modification‐related enzymes (Figure S6f–n, Supporting Information). Furthermore, PXR knockdown abolished the promoting effect of IPA on FTO protein expression (Figure 6h,j), suggesting that PXR specifically regulates the expression of FTO.

As a classic nuclear transcription factor, we hypothesized that PXR could transcriptionally regulate FTO expression, thereby modulating the overall m^6^A modification level in hepatocytes. We predicted and analyzed the PXR protein binding site in the FTO promoter sequence and identified one potential PXR binding site (Figure 6k, Table S4, Supporting Information). Using ChIP‐PCR technology, we observed a significant decrease in PXR protein binding abundance at the potential binding site in the FTO promoter sequence after DON treatment, which was significantly increased after PXR agonist IPA treatment (Figure 6l). IPA pretreatment counteracted the decrease in PXR binding abundance caused by DON (Figure 6l). Additionally, we validated the functionality of these binding sites using the dual‐luciferase reporter system. Deletion of the binding site in the FTO promoter prevented DON‐induced suppression and IPA‐induced alleviation of the FTO gene promoter (Figure 6m,n). These results indicate that PXR directly activates the transcription of the FTO gene.

To demonstrate the regulatory role of PXR transcriptionally activated FTO on the expression level of Malat1 lncRNA in hepatocytes and DON‐induced oxidative stress, we overexpressed FTO and observed a significant reduction in the upregulated expression of Malat1 lncRNA induced by DON in hepatocytes (Figure 6o). Additionally, FTO overexpression alleviated the expression imbalance of oxidative stress‐related enzymes MDA/GSH caused by DON (Figure 6p,q) and the excessive generation of ROS (Figure 6r,s) in cells. In summary, our findings reveal that PXR transcriptionally activates FTO expression, leading to the regulation of m^6^A modification and expression levels of Malat1 lncRNA in hepatocytes, ultimately modulating the DON‐induced oxidative stress process.

### 2.7 Malat1 m^6^A Modification Recruited YTHDC1 to Promote its Stability and Expression

**Figure 7:**
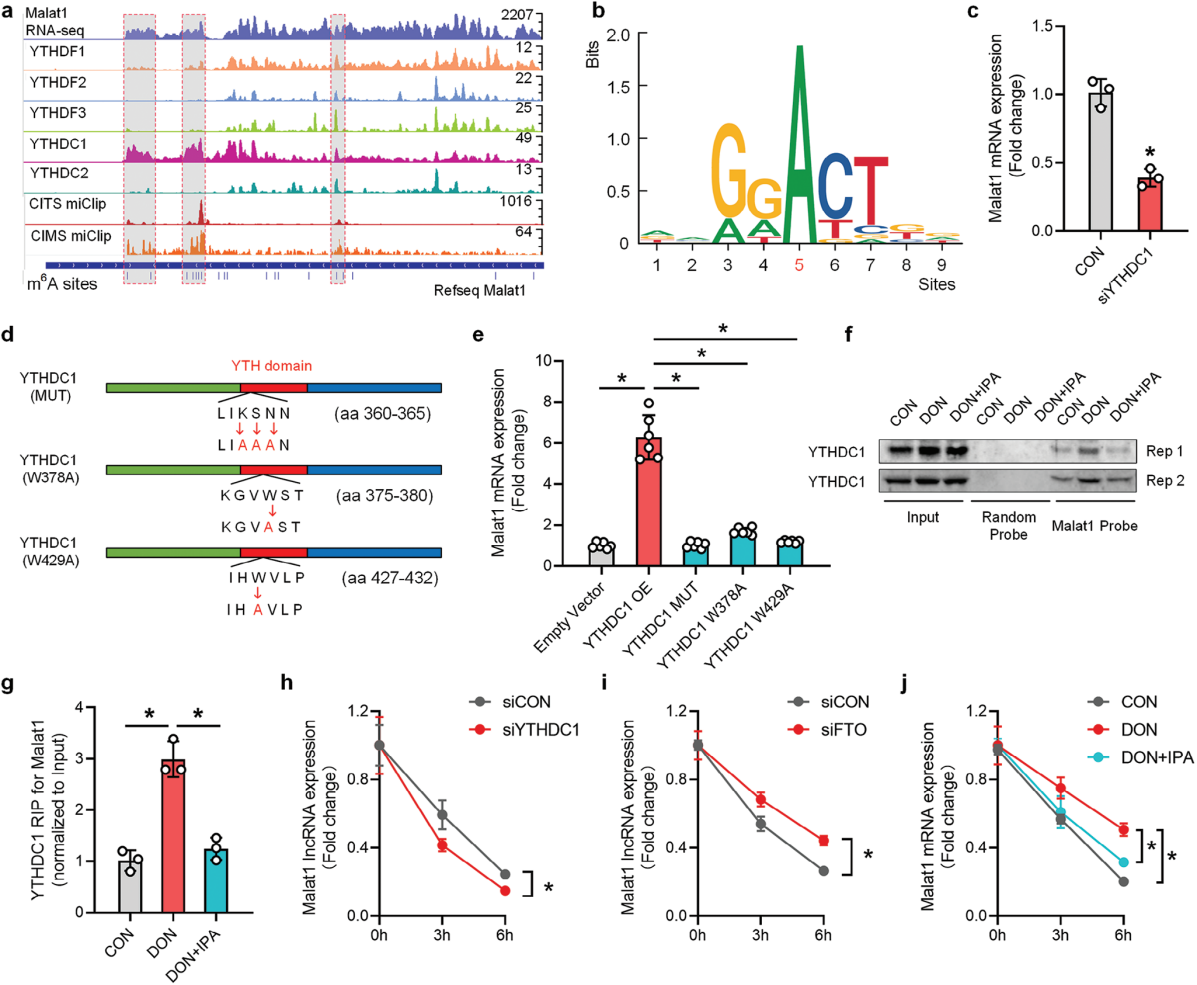
Malat1 m^6^A modification recruits YTHDC1 to promote its stability and expression. a) Evaluation and determination of endogenous YTH proteins that preferentially interact with m^6^A sites within the Malat1 transcriptome range were carried out through published RNA‐seq (GSE190324) and iCLIP‐seq (GSE78030, GSE63753) databases. High‐density Malat1 m^6^A modification regions were represented by gray shading. The Y‐axis represents the read counts of the respective genes obtained from sequencing. The numbers indicate the read counts of the highest peak in the corresponding peak graphs. b) Characteristic motifs of YTHDC1 protein binding to Malat1 lncRNA. c) Expression levels of Malat1 lncRNA in AML12 cells treated with YTHDC1 knockdown (n = 3). d) Schematic diagram showing the position of m^6^A binding site mutations in the YTH domain of YTHDC1 protein. aa, amino acid. e) Expression levels of Malat1 lncRNA when transfected with YTHDC1 overexpression plasmid and YTH m^6^A binding site mutation plasmid (n = 6). f,g) RNA affinity purification (n = 2) and RIP‐qPCR (n = 3) analysis of the interaction between YTHDC1 protein and Malat1 mRNA. h) Knockdown of YTHDC1 in AML12 cells were treated with 5 µg mL^−1^ actinomycin D for 0, 3, and 6 h. The expression levels of Malat1 mRNA were analyzed by RT‐qPCR (n = 3). i) Knockdown of FTO in AML12 cells were treated with 5 µg mL^−1^ actinomycin D for 0, 3, and 6 h. The expression levels of Malat1 mRNA were analyzed by RT‐qPCR (n = 3). j) AML12 cells were treated with 100 µmol L^−1^ IPA for 12 h, then treated with 10 nmol mL^−1^ DON for 12 h, followed by treatment with 5 µg mL^−1^ actinomycin D for 0, 3, and 6 h. The expression levels of Malat1 mRNA were analyzed by RT‐qPCR (n = 3). Values are expressed as mean ± standard deviation, *P < 0.05.

To elucidate the mechanism underlying the regulation of Malat1 lncRNA transcription by m^6^A residues, we investigated the interactions between m^6^A readers and Malat1 lncRNA. YTHDF1/2/3 and YTHDC1/2 are known m^6^A readers that can recognize and bind m^6^A residues. While YTHDF1/2/3 and YTHDC2 primarily localize in the cytoplasm, YTHDC1 is predominantly found in the nucleus.^[^ ^23^ ^]^ Considering that Malat1 lncRNA is expressed and functions in the nucleus, we hypothesized that nuclear m^6^A reader YTHDC1 is likely to recruit and bind to Malat1 lncRNA. To assess the binding properties of YTH proteins with Malat1 lncRNA m^6^A residues, we analyzed published RNA‐seq (GSE190324) and iCLIP‐seq (GSE78030, GSE63753) databases (**Figure** **7**a). The results revealed that only YTHDC1 exhibited a clear preference for binding to the entire set of Malat1 m^6^A residues (Figure 7a). Most of the determined narrow peaks exhibited the classic consensus sequence RRm^6^ACU (R = A/G) (Figure 7b).

To demonstrate the dependence of Malat1 lncRNA expression on YTHDC1, we transfected AML12 cells with YTHDC1 siRNA and observed a significant inhibition of Malat1 lncRNA expression (Figure 7c). Additionally, we constructed three mutants of YTHDC1 known to have impaired m^6^A‐binding capacity and transfected them into AML12 cells (Figure 7d). The results showed that only the overexpression of wild‐type YTHDC1 (YTHDC1 OE) upregulated Malat1 lncRNA expression, while overexpression of YTH m^6^A‐binding mutants of YTHDC1 had no effect on Malat1 expression (Figure 7e), indicating that Malat1 lncRNA expression is initiated in an m^6^A‐ and YTHDC1‐dependent manner.

We further analyzed the interaction between Malat1 lncRNA and YTHDC1 protein through Malat1 lncRNA affinity‐isolation and YTHDC1 RIP‐qPCR analysis. The results demonstrated that YTHDC1 protein could be affinity‐isolated with a Malat1 probe (Figure 7f), and Malat1 lncRNA could be pull‐down by YTHDC1 antibody (Figure 7g). Moreover, under DON treatment, the binding abundance between Malat1 and YTHDC1 significantly increased, while IPA pretreatment attenuated the DON‐induced upregulation of Malat1 lncRNA‐YTHDC1 protein binding abundance (Figure 7f,g). YTHDC1 has been shown to enhance the stability of target RNA,^[^ ^24^ ^]^ and our findings were consistent with this, knockdown of YTHDC1 markedly reduced the stability of Malat1 lncRNA (Figure 7h). Conversely, knockdown of FTO increased the m^6^A modification level of Malat1 lncRNA (Figure S6o, Supporting Information), resulting in the recruitment of more YTHDC1 and enhanced stability of Malat1 lncRNA (Figure 7i). In the hepatocyte oxidative stress model induced by DON, the hyper‐m^6^A modification of Malat1 increased its dependence on YTHDC1‐mediated RNA stability. Treatment with the PXR activator IPA reduced the m^6^A modification level of Malat1 lncRNA by upregulating the expression of demethylase FTO, thereby suppressing the DON‐induced high stability of Malat1 (Figure 7j). Overall, these data indicate that m^6^A modification recruits YTHDC1 to promote the stability and expression of Malat1 lncRNA. The excessive binding of Malat1 lncRNA to NRF2 protein inhibits its activity, ultimately resulting in significantly suppressed antioxidant capacity and increased oxidative stress in hepatocytes.

### 2.8 IPA and Malat1 Knockout Alleviated DON‐Induced Oxidative Stress and Liver Injury In Vivo

**Figure 8:**
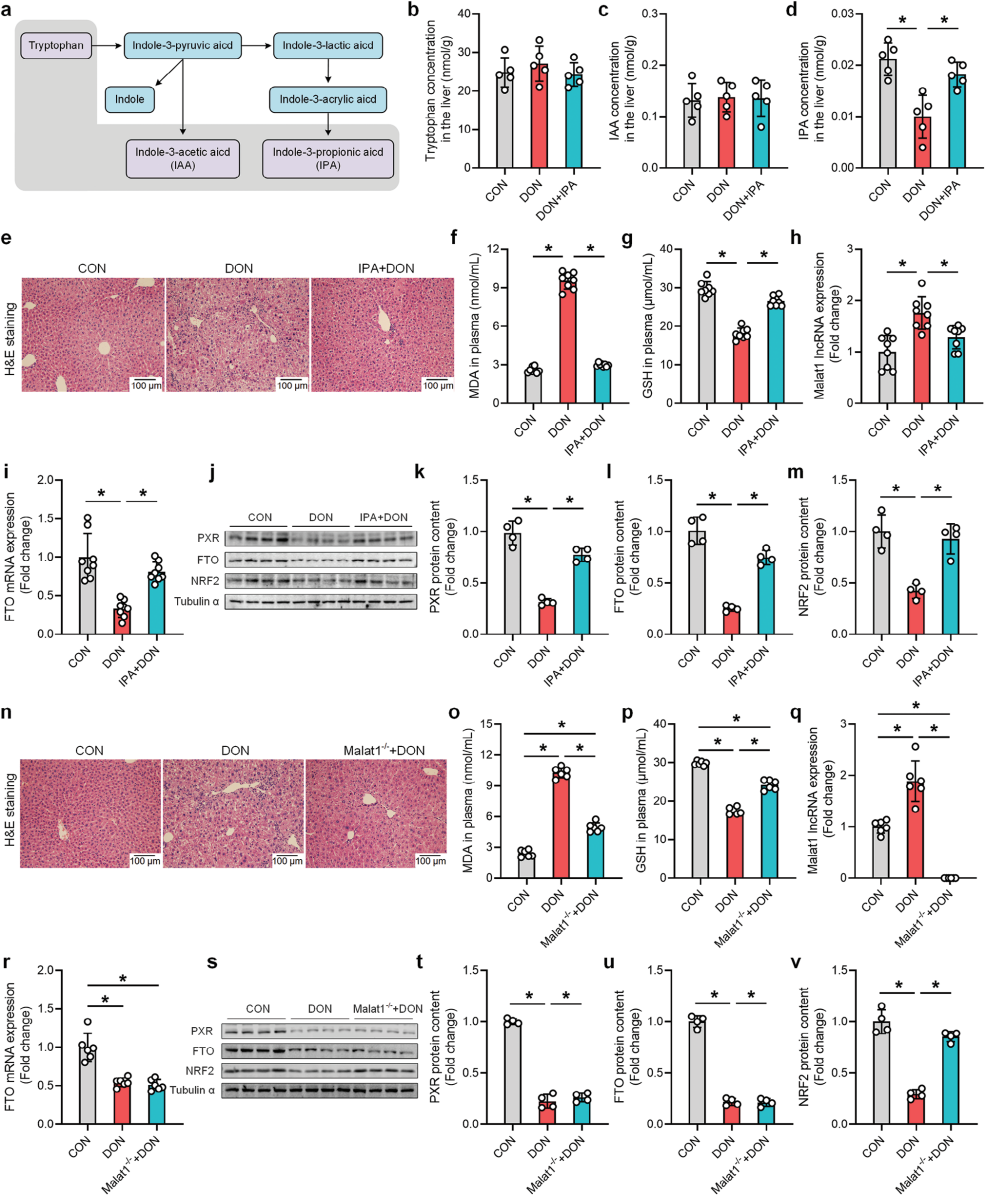
IPA and knockout of Malat1 alleviated DON‐induced oxidative stress and liver injury in mice. a) Tryptophan is metabolized by gut microbiota to produce metabolites, mainly including IAA and IPA. b–d) LC‐MS was used to detect the content of tryptophan, IAA and IPA in the liver after treatment with DON and IPA (n = 5). e) HE staining investigated the alleviating effect of IPA treatment on DON‐induced liver injury in mice. f, g) IPA treatment alleviated the disorder of MDA and GSH content, key enzymes of liver oxidative stress induced by DON (n = 8). h) IPA treatment alleviated the elevated expression of Malat1 lncRNA induced by DON in the liver (n = 8). i) IPA treatment alleviated the decreased expression of FTO mRNA induced by DON in the liver (n = 8). j–m) IPA treatment alleviated the decreased expression of PXR, FTO and NRF2 proteins induced by DON in the liver (n = 4). n) HE staining investigated the alleviating effect of Malat1 knockout on DON‐induced liver injury in mice. o,p) Malat1 knockout alleviated the disorder of MDA and GSH content, key enzymes of liver oxidative stress induced by DON (n = 8). q) Malat1 knockout alleviated the elevated expression of Malat1 lncRNA induced by DON in the liver (n = 8). r) Malat1 knockout alleviated the decreased expression of FTO mRNA induced by DON in the liver (n = 4). s–v) Malat1 knockout alleviated the decreased expression of PXR, FTO and NRF2 proteins induced by DON in the liver (n = 4). Values are expressed as mean ± standard deviation, *P < 0.05.

To validate the regulatory role of the PXR‐FTO‐Malat1 lncRNA‐NRF2 pathway and explore potential therapeutic strategies for DON‐induced liver injury, we conducted in vivo experiments in mice (Figure S7a, Supporting Information). Our results showed that the concentration of IPA, the bacterial metabolite of tryptophan (**Figure** **8**a), was significantly reduced in the livers of mice treated with DON (Figure 8d). However, pretreatment with IPA effectively alleviated this effect (Figure 8d), suggesting that supplemental IPA could be a potential therapeutic strategy against DON toxicity. Neither IPA nor DON treatment had any effect on the concentrations of tryptophan or its metabolite, indole‐3‐acetic acid (IAA), in the liver (Figure 8b,c). Hematoxylin and eosin (H&E) staining of liver sections revealed that DON induced inflammatory cell infiltration and caused hepatocyte damage (Figure 8e). Blood biochemical indicators associated with liver injury (AST, ALT; Figure S7b,c, Supporting Information) and oxidative stress (MDA, GSH; Figure 8f,g) were abnormal, and the expression levels of genes related to antioxidant pathways in the liver were significantly decreased (Figure 8j,m; Figure S7e–i, Supporting Information). However, pretreatment with IPA effectively attenuated these effects, indicating that IPA pretreatment can significantly alleviate DON‐induced liver injury and oxidative stress in mice. Furthermore, we observed that DON treatment significantly inhibited the expression of PXR (Figure 8j,k; Figure S7d, Supporting Information) and its downstream factor FTO (Figure 8i,j,l). However, PXR pretreatment significantly mitigated this downregulation at both the mRNA and protein levels, subsequently alleviating the overexpression of Malat1 regulated by m^6^A modification (Figure 8h).

**Figure 9:**
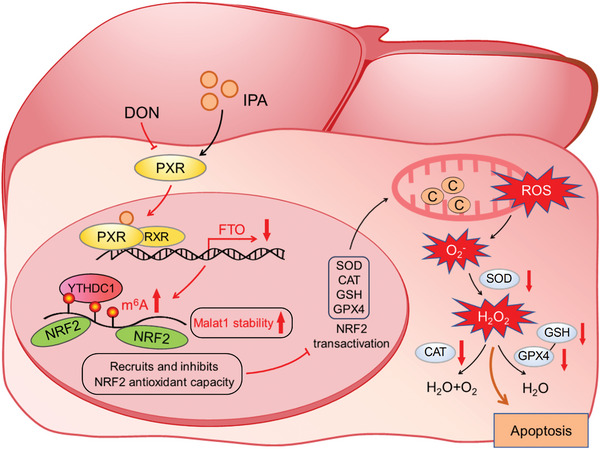
In the model of DON‐induced liver damage and oxidative stress in hepatocytes, treatment with the PXR agonist IPA activates FTO, an m^6^A demethylase. This activation leads to site‐specific demethylation of Malat1 lncRNA and reduces the abundance of YTHDC1‐bound Malat1 lncRNA. The m^6^A demethylation of Malat1 enhances its stability and augments the NRF2‐controlled antioxidant pathway, ultimately mitigating DON‐induced liver injury.

Similar results were obtained in Malat1 knockout mice (Figure 8q) treated with DON (Figure S7j, Supporting Information). Malat1 knockout significantly alleviated DON‐induced liver injury in mice, as evidenced by significant improvements in H&E staining (Figure 8n), blood biochemical indicators (Figure 8o,p; Figure S7k,l, Supporting Information), and the expression of antioxidant‐related genes (Figure S7n–r, Supporting Information). However, Malat1 knockout did not affect the DON‐induced decrease in hepatic PXR and FTO expression (Figure 8r–v; Figure S7m, Supporting Information). Overall, these results highlight the critical role of Malat1 lncRNA in DON‐induced liver injury and oxidative stress, validating the regulatory role of the PXR‐FTO‐Malat1 lncRNA‐NRF2 pathway in DON‐induced liver injury in vivo (**Figure** **9**). These findings provide a foundation for the development of novel therapeutic interventions targeting this pathway to prevent or treat DON‐induced liver injury.

## 3 Discussion

PXR is a pivotal regulator in the detoxification of xenobiotics by binding to specific response elements within gene regulatory regions. Through its binding activity, PXR can modulate downstream gene expression, influencing various processes such as detoxification, metabolism, inflammation inhibition, cell apoptosis, tumor migration, and antioxidant stress.^[^ ^22^, ^25^ ^]^ The effects of PXR in diseases such as drug‐induced liver injury and cancer are multifaceted, exhibiting both positive and negative effects depending on the context. Positive effects include detoxification, anti‐inflammatory and anti‐apoptotic effects, and lifespan extension,^[^ ^26^ ^]^ while negative effects involve increased expression of drug‐metabolizing enzymes leading to reactive metabolite formation, hyperglycemia, lipid accumulation, and steatosis.^[^ ^27^ ^]^ In this study, we demonstrated that activation of PXR effectively alleviates hepatic oxidative stress and apoptosis induced by xenobiotic DON, highlighting the crucial role of targeting PXR activation in combating xenobiotic pollution and enhancing hepatic antioxidant capacity.

Malat1 lncRNA has been implicated in the pathogenesis of various diseases, including cancer, diabetes, non‐alcoholic fatty liver disease, inflammatory response, and neurodegenerative diseases.^[^ ^28^ ^]^ Excessive cellular oxidative stress is a common characteristic of these diseases, and Malat1 lncRNA has been shown to play a regulatory role in oxidative stress responses.^[^ ^29^ ^]^ Consistent with previous reports,^[^ ^30^ ^]^ hepatic oxidative stress plays a significant role in DON toxicity and liver damage. In this study, RNA‐seq analysis revealed that DON‐treated hepatocytes exhibited enriched oxidative stress‐related pathways, accompanied by downregulation of NRF2‐mediated antioxidant‐related transcripts, such as GSTA1/O1, CAT, SOD, and GPX4. Knockdown of Malat1 both in vitro and in vivo, as well as pretreatment with the PXR agonist IPA, mitigated these effects. Furthermore, RNA immunoprecipitation revealed that the NRF2 protein binds to Malat1 lncRNA in the nucleus of hepatocytes. Under DON treatment, the expression level of Malat1 lncRNA increased, leading to a significant increase in its binding to the NRF2 protein, consequently impairing NRF2's transcriptional activation and expression of antioxidant‐related molecules. This impairment severely compromised the antioxidant capacity of hepatocytes. These findings are consistent with our previous discoveries^[^ ^11^ ^]^ that Malat1 lncRNA acts as a negative regulator of the NRF2 protein, thereby contributing to cellular oxidative stress.

Hyper‐m^6^A methylation of Malat1 lncRNA has been demonstrated to enhance its stability and promote cancer cell proliferation and migration.^[^ ^20^ ^]^ Our investigation unveiled that DON treatment significantly elevated the m^6^A modification levels at specific sites (1206, 1388 nt) on Malat1 lncRNA. Subsequent analysis utilizing RNA‐pulldown and RNA immunoprecipitation techniques elucidated that DON treatment enhances the binding of Malat1 lncRNA to the m^6^A‐modified protein YTHDC1, thereby augmenting the stability and expression levels of Malat1 lncRNA. YTHDC1 is known to recognize m^6^A modification sites and participate in the degradation of m^6^A‐marked chromatin‐associated regulatory RNAs (carRNAs) through the nuclear exosome targeting (NEXT) complex‐mediated nuclear degradation mechanism.^[^ ^31^ ^]^ Moreover, YTHDC1 participates in the assembly of ribonucleoprotein complexes, wherein RNAs and proteins coalesce to form functionally ordered complexes that participate in biological processes during early embryonic development.^[^ ^32^ ^]^ Following m^6^A modification, certain nuclear lncRNAs can be bound by YTHDC1, thereby influencing transcription. For instance, m^6^A residues on XIST, an lncRNA responsible for transcriptional silencing of the entire X chromosome, recruit YTHDC1 to form a transcriptional silencing complex, consequently facilitating XIST‐mediated transcriptional repression.^[^ ^33^ ^]^ Similarly, m^6^A modification on HEAT lncRNA can recruit YTHDC1, leading to the silencing of stress genes during the recovery phase of heat shock to alleviate the heat shock response.^[^ ^34^ ^]^ Specific m^6^A modification sites on HOTAIR lncRNA promote the binding to YTHDC1, facilitating the interaction of HOTAIR with target chromatin and gene suppression, ultimately promoting the proliferation and invasion of breast cancer cells.^[^ ^35^ ^]^ In our study, we provide the first comprehensive analysis of DON treatment‐induced significant increase at the specific m^6^A modification sites on Malat1 lncRNA with single‐base resolution and recruitment of YTHDC1, enhancing the stability and expression levels of Malat1 lncRNA. Additionally, the increased association between the NRF2 protein and Malat1 lncRNA resulted in the inhibition of antioxidant‐related gene expression.

Various ligands and activators have been identified upstream of PXR, including rifampicin, vitamin E, IPA, arsenic, bile acids, and other chemicals such as SPA70, pregnenolone 16α‐carbonitrile (PCN), and alismanin A.^[^ ^22^ ^]^ However, in the context of hepatic oxidative stress and injury caused by exogenous substances and environmental pollutants, it is crucial to selectively activate PXR while minimizing any impact on other nuclear receptors (such as CAR and LXR) and ensuring well‐established pharmacokinetics and safety profiles of potential drug candidates. To screen for promising drug candidates, we selected rifampicin or IPA, which are known classical activators of PXR. Our results revealed that both rifampicin and IPA can activate the PXR‐mediated FTO‐m^6^A‐Malat1 pathway, thereby mitigating DON‐induced oxidative stress in hepatocytes. Notably, IPA exhibited superior efficacy compared to rifampicin in activating PXR and reducing oxidative stress. This difference in efficacy may be attributable to rifampicin's weaker activity on mouse PXR and its classification as a human PXR activator. Although direct comparison of the effects of rifampicin and IPA on oxidative stress in human hepatocytes was limited by experimental constraints, we observed significantly lower IPA levels in the livers of mice with DON‐induced oxidative stress and liver injury compared to the control group, while tryptophan and IAA levels remained unchanged. IPA, a secondary metabolite of tryptophan derived from intestinal bacterial metabolism, can be absorbed into the bloodstream through the intestine, thus increasing its content in the liver upon supplementation and mitigating DON‐induced liver oxidative stress. These findings underscore the significance of IPA in the context of liver injury and oxidative stress caused by DON, providing strong support for its potential clinical application in the treatment of liver oxidative stress and injury resulting from xenobiotics or environmental pollutants. Furthermore, these results highlight the crucial role of the gastrointestinal tract, as the primary organ exposed to xenobiotics or environmental pollutants, in targeting and mitigating oxidative stress and cellular damage, including disruptions to gut microbiota and metabolite profiles.^[^ ^36^ ^]^

Our research focuses on investigating the oxidative damage caused by DON in the liver. Unlike ruminant animals’ rumen or birds’ crops and cecum, the intestinal microbiota of humans lack specific microbes,^[^ ^37^ ^]^ such as *Eubacterium* BBSH797 and *Eggerthella* sp. DII‐9, that convert DON into the less toxic form known as de‐epoxy DON (DOM‐1).^[^ ^38^ ^]^ Consequently, in humans, DON is absorbed by the intestinal epithelial cells through simple diffusion and rapidly metabolized in the liver through glucuronidation processes, resulting in the formation of metabolites such as DON‐3‐glucuronide and DON‐15‐glucuronide.^[^ ^39^ ^]^ These metabolites, which are generally less toxic, are eliminated through the kidneys via urine excretion.^[^ ^4^ ^]^ However, prolonged exposure to DON can disrupt liver detoxification function, leading to oxidative damage. While enhancing the liver's antioxidant capacity is recognized as a viable strategy to mitigate the detrimental effects of DON exposure,^[^ ^5^, ^6^, ^40^ ^]^ it is important to acknowledge that prolonged exposure to DON not only damages the liver but also significantly impacts the intestines. Research suggests that sustained DON exposure can disrupt intestinal barrier function, increase intestinal permeability, and compromise the integrity of the intestinal epithelial layer.^[^ ^41^ ^]^ This disruption facilitates the translocation of toxins, pathogens, and other harmful substances from the intestinal lumen into the bloodstream, triggering inflammatory responses and contributing to systemic health issues. Furthermore, prolonged DON exposure can alter the composition of the gut microbiota, which is associated with various intestinal disorders and systemic health problems.^[^ ^42^ ^]^ Therefore, future research endeavors will focus on elucidating the mechanisms underlying DON‐induced damage to the gut‐microbiota‐liver axis and exploring novel strategies for drug development.

## 4 Conclusion

In summary, our study reveals that prolonged exposure to DON suppresses PXR expression, resulting in the repression of m^6^A demethylase FTO and increased m^6^A modification on Malat1 lncRNA. This disruption impairs NRF2‐mediated antioxidant pathways, leading to heightened oxidative stress in hepatocytes. Importantly, we demonstrate the therapeutic potential of the PXR agonist IPA in mitigating xenobiotic‐induced liver injury by restoring antioxidant capacity. Our findings highlight the crucial role of the newly discovered PXR‐FTO‐Malat1 lncRNA m^6^A‐NRF2 signaling pathway and provide valuable insights for developing targeted interventions. Further research is needed to explore clinical applications and additional mechanisms underlying xenobiotic DON‐induced liver injury. Overall, our study contributes to advancing our understanding of hepatic pathophysiology and offers promising prospects for the development of effective treatments for DON‐induced liver injury associated with oxidative stress.

## 5 Experimental Section

### Cell Culture and Treatment

According to a previously published protocol,^[^ ^16b^ ^]^ mice were euthanized, and primary hepatocytes (HC) were isolated from the liver. The AML12 mouse hepatocyte cell line (CRL‐2254, ATCC, USA) and primary HC were cultured in DMEM/F12 medium (SH30023.01, Hyclone, USA) supplemented with 10% fetal bovine serum (16140071, Gibco, USA), 1× ITS Liquid Media Supplement (100×, C0345, Beyotime, China), and 40 ng mL^−1^ dexamethasone (Id0170, Solarbio, China) at 37 °C in a 5% CO_2_ incubator. The cells were allowed to reach 60% confluency before being treated with 100 nmol mL^−1^ of IPA (830‐96‐6, Aladdin, China) and 10 nmol mL^−1^ of Rifampin (RIF, R8011, Solarbio, China) for 12 h, followed by an additional 12 h exposure to 10 nmol mL^−1^ of DON (B135944, Aladdin, China).

### CCK‐8 Cell Viability Assay

To evaluate cell activity, AML12 cells and primary HC were treated with varying concentrations of IPA/RIF and cultured for 4 h. Following a 2 h incubation with CA1210 reagent (Solarbio, China), the absorbance of the cells was measured at 450 nm using a microplate reader (Biotek, USA).

### Mice and Treatment

Malat1 knockout mice were purchased from the Texas A&M Institute for Genomic Medicine (TIGM). Male wild‐type C57BL/6 mice and Malat1 knockout mice aged 7 weeks (Issue No. AW70012202‐1‐1) were randomly divided into the control (CON) group, deoxynivalenol group (DON), and Malat1 knockout with deoxynivalenol (gavage) group (Malat1 KO+DON). 7‐weeks‐old C57BL/6 male mice (Issue No. AW70012202‐1‐1) were randomly divided into the control (CON) group, deoxynivalenol group (DON), and IPA with deoxynivalenol (gavage) group (IPA+DON). Each group has four replicates, and every replicate has three mice. The mice were housed in standard conditions of 23±2 °C and 50±10% relative humidity, light‐dark cycle for 12 h, and free access to food and water. They were acclimatized to the new environment for two weeks.

According to a previous study, IPA (830‐96‐6, Aladdin, China) was dissolved and prepared into 1 mg mL^−1^ in sterile water.^[^ ^43^ ^]^ DON was dissolved in sterile water, with the concentration used according to previous studies to ensure that male mice could survive during the experiment. For treatment, the mice were first pretreated with IPA for 15 days (Day 1‐Day 15). The control group and the DON group were given 150 µL time^−1^ of normal saline by gavage. The IPA group was given 150 µL of 10 mg mL time^−1^ IPA by gavage every 2 days. After pretreatment (Day 15), the mice were co‐treated with DON for 15 days. Based on the original treatment, the DON group, Malat1 KO+DON group, and IPA+DON group were additionally gavaged with 2 mg k^−1^g time^−1^ DON every 2 days. For sampling, blood was withdrawn from the retro‐orbital venous plexus, mice were euthanized by cervical dislocation, and the livers were harvested. The procedures for this experiment were carried out in accordance with the guidelines set by China Agricultural University's animal protection and utilization organization committee (CAU20171015‐3).

### Caspase‐1 and Caspase‐3 Activity Assays

To extract proteins from hepatocytes and minced liver tissue, they were immersed in RIPA solution and subjected to cell lysis. The protein concentration was determined and calibrated using the BCA protein assay kit (23 225, Thermo Fisher, USA). The enzymatic activities of caspase‐1 (C1102, Beyotime, China) and caspase‐3 (C1115, Beyotime, China) were measured using the Caspase Activity Assay Kit. The optical absorption at a wavelength of 405 nm was detected using a microplate reader (Biotek, USA).

### Flow Cytometric Analysis of Apoptosis and ROS

Measurement of cell apoptosis was performed using the Annexin V‐FITC/PI Apoptosis Detection Kit (CA1020, Solarbio, China). AML12 cells and primary HC were incubated with Annexin V‐FITC and PI dye, and the fluorescent signals were detected using flow cytometry. For the detection of cellular ROS levels, the CellROX Green Reagent (C10444, Invitrogen, USA) was utilized following the manufacturer's instructions. CellROX dye was added to each well, and after incubation in the dark with vigorous mixing, cells were harvested and washed with HBSS buffer. The CellROX fluorescence was measured using flow cytometry.

### Analysis of MDA Content and GSH Activity

The quantification of Malondialdehyde (MDA) content in liver tissue, primary HC, and AML12 cells was performed using the Thiobarbituric Acid (TBA) spectrophotometric kit (MDA‐TBA kit) (BC0025, Solarbio, China). The optical absorption at 532 nm was measured using a Biotek microplate reader. GSH‐Px activity in liver tissue and AML12 cells was measured using the GSH‐Px ELISA kits (BC1170, Solarbio, China), and the optical absorption wavelength was detected at 412 nm using a microplate reader (Biotek, USA).

### Protein Extraction and Western Blot

Total protein was isolated using RIPA buffer, and cytoplasmic and nuclear proteins were extracted using the Nuclear and Cytoplasmic Protein Extraction kit (P0027, Beyotime, China), then quantified with the BCA Protein Assay kit (23 225, Thermo Fisher, USA). Subsequently, 20 µg of proteins were subjected to SDS‐PAGE electrophoresis using 10% or 15% gels and transferred onto a nitrocellulose membrane. The following primary antibodies were utilized: anti‐FTO (27226‐1‐AP, 1:1000, Proteintech, China), anti‐PXR (67912‐1‐Ig, 1:1000, Proteintech, China), anti‐YTHDC1 (ab122340, 1:1000, Abcam, USA), and anti‐NRF2 (16396‐1‐AP, 1:1000, Proteintech, China), with Tubulin α (K006154P, 1:1000, Solarbio, China) selected as an internal control. Images were captured by the Odyssey infrared imager (LI‐COR, USA), and band density was analyzed using Quantity One software (Bio‐Rad, USA).

### RNA Isolation and qPCR

Total RNA was isolated from the liver and hepatocytes using TRIzol Reagent (TSP401, Tsingke Biotech, China). Subsequently, 1 µg of total RNA was reverse transcribed into cDNA using the HiScript II Reverse Transcriptase kit (R233‐01, Vazyme, China). RT‐qPCR was performed on the LightCycler 96 Instrument (Roche, Switzerland) using 2 µL of diluted cDNA (1:20, v:v). All primers (Table S1, Supporting Information) were synthesized by Tsingke Biotech (Beijing, China), and PPIA was selected as the reference gene due to its stability under the treatment conditions.

### Immunofluorescence

AML12 cells were fixed in 4% paraformaldehyde and washed with PBS buffer. To disrupt the cell membrane, they were soaked in Tris‐buffered saline containing Triton X‐100 (0.3%) for 1 h, then blocked with 10% goat serum for 1 h. Overnight incubation at 4 °C was carried out with PXR antibody (67912‐1‐Ig, Proteintech, China, 1:300) and NRF2 antibody (16396‐1‐AP, Proteintech, China, 1:300), followed by incubation with a fluorescent secondary antibody. DAPI dye was utilized to label the nuclei.

### Dual‐Luciferase Reporter Assay

The sequence of the FTO promoter fragment (2000 bp) was obtained from the Ensembl gene database (http://asia.ensembl.org/index.html) and chemically synthesized by Tsingke Biotech (Beijing, China). The fragment was then ligated into the pGL3‐basic vector plasmid (Promega, USA) between XhoI and HindIII sites, resulting in the FTO promoter WT construct. Additionally, the predicted PXR binding site in the FTO promoter fragment was deleted and chemically synthesized by Tsingke Biotech, and then cloned into the pGL3‐basic vector plasmid, generating the FTO promoter MUT construct. Luciferase activity was measured using a GloMax 96 Microplate Luminometer (Promega, USA), and the ratio of firefly luciferase activity to Renilla luciferase activity was used to determine relative luciferase activity.

### Plasmid/siRNA Construction and Cell Transfection

pRlenti‐3×Flag vector (Empty vector), pRlenti‐3×Flag‐YTHDC1 (YTHDC1 OE), pRlenti‐3×Flag‐ YTHDC1‐Mutation (YTHDC1 MUT), pRlenti‐3×Flag‐YTHDC1‐W378A (YTHDC1 W378A), and pRlenti‐3×Flag‐YTHDC1‐W429A were kindly provided by Prof. Jiekai Chen. pEX‐1‐FTO overexpression plasmid was provided by Genepharma Biotechnology Co., Ltd. in Suzhou, China. Cells were transfected using the jetPRIME reagent (101 000 046, Polyplus, USA). Lentivirus was generated using HEK293T cells. Specifically, when HEK293T cells reached ≈80% confluency, the pRlenti vector containing the target gene was co‐transfected with psPAX2 and pMD.2G plasmids (packaging plasmids). The viral supernatants were collected and pooled at 48 and 72 h post‐transfection, and Lentivirus was obtained by filtering the viral supernatants through a 0.45 mm filter (Millipore, USA). Lentivirus and siRNA (Tsingke, China) were transfected when the confluency of AML12 cells was ≈60%. The siRNA sequences were listed in Table S2 (Supporting Information).

### RNA Decay Assay

AML12 cells were cultured and treated with or without DON/IPA followed by the addition of actinomycin D (5 µg mL^−1^, HY‐17559, MCE, USA) to each cell. After 0, 3, and 6 h, cells were collected, and RNA was extracted for RT‐qPCR analysis to determine the relative abundance of Malat1 lncRNA (compared to 0 h).

### ChIP‐PCR Assay

AML12 cells were cultured in 10 cm dishes and treated with 1% formaldehyde solution for cross‐linking, followed by quenching with 125 mmol L^−1^ glycine. The cells were then scraped off, collected by centrifugation, and washed with cold PBS buffer. To generate DNA fragments, coarse chromatin preparations were sonicated after losing the cells with sodium dodecyl sulfate lysis buffer containing a protease inhibitor cocktail (HY‐K0010, MCE, USA). Anti‐PXR antibody (67912‐1‐Ig, Proteintech, China) was incubated overnight at 4 °C with pre‐cleared chromatin preparations, while normal IgG (2729S, Cell Signaling Technology, USA) was used as the negative control. Immunoprecipitated chromatin complexes were captured using agarose beads containing Protein A/G. Finally, the immunoprecipitated complexes were subsequently reverse cross‐linked for 1 h at 65 °C to release the DNA fragments and quantified using quantitative real‐time PCR with specific primers for fragments of the FTO gene promoter. The primer sequences were listed in Table S1 (Supporting Information).

### RNA m^6^A Dot Blot Assay

A 500 ng uL^−1^ concentration of RNA was calibrated and denatured at 95 °C for 5 min, then accurately pipetted and dropped onto a Hybond‐N^+^ membrane. TBST buffer was used to wash the membrane after UV cross‐linking for 10 min, non‐fat milk was used for blocking, and anti‐m^6^A antibody (68055‐1‐Ig, Proteintech, China) was incubated overnight with the membrane at 4 °C. The membrane was subsequently soaked in secondary antibodies and incubated for 2 h at room temperature. Images were captured using an Odyssey infrared imager (LI‐COR, USA), and band density was analyzed using Quantity One software (Bio‐Rad, USA). To control loading, RNA dot blots were stained with 0.02% methylene blue buffer (0.3 mol L^−1^ NaOAc, pH = 5.2) prior to blocking with non‐fat milk.

### LC‐MS/MS for Determination of the m^6^A/A Ratio

UPLC‐ESI‐QQQ analysis was conducted using a C18 column (Rapid Resolution HT, 50 mm×2.1 mm I.D, 1.8 µm; Agilent, USA). The mobile phase consisted of formic acid in methanol (0.1% v/v, solvent B) and formic acid in water (0.1% v/v, solvent A). Mass spectrometry analysis was performed, and production scans were obtained for protonated m6A at m/z (282 → 150) and A at m/z (268 → 136). For the preparation of RNA samples, ZnCl2 (20 mmol L^−1^), NaCl (100 mmol L^−1^), and nuclease P1 (1 µL, N8630‐1Vl, Sigma–Aldrich, USA) were added to 500 ng of RNA in a total volume of 25 µL. The mixture was incubated at 37 °C for 2 h. Afterward, the solution was diluted to a volume of 1 mL with deionized water and filtered through a 0.22 µm Millipore membrane. Five microliters of the resulting solution were injected into the LC‐MS/MS system for detection. To quantify the m^6^A and A content in the samples, m^6^A and A standards ranging from 0.1–10 nmol L^−1^ for m^6^A and 50–2000 nmol L^−1^ for A were simultaneously analyzed to generate a standard curve. The m^6^A levels were calculated as the ratio of m^6^A to A.

### LC‐MS/MS for Determination of Tryptophan, Indole‐3‐Acetic Acid, and Indole‐3‐Propionic Acid Content

A 100 mg liver sample was homogenized in 1 mL of a 50:50 mixture of methanol and molecular‐grade water at 4 °C for three cycles. After allowing the sample to stand for 15 min, it was centrifuged at 10 000 g for 5 min, and the supernatant was collected and filtered through a 0.22 µm filter (Millipore, USA). Subsequently, 500 µL of the filtered supernatant was pipetted into a glass tube for LC‐MS analysis. For the LC‐MS analysis, a C18 column (3.0 mm × 150 mm, 1.8 µm) was employed, coupled with an Agilent 1260 SFC‐Ultivo system and an Agilent 1290 UPLC electrospray ionization‐time‐of‐flight mass spectrometer (ESI‐TOF‐MS). The eluents consisted of water with 0.1% formic acid (eluent A) and 100% methanol (eluent B), which were mixed to create a linear gradient. The analysis was performed at a flow rate of 0.3 mL min^−1^, and the column temperature was maintained at 40 °C. The following elution gradients were utilized: 0‐0.5 min, 20% eluent B; 0.5–1 min, 20%–40% eluent B; 1–2 min, 40%–50% eluent B; 2–3 min, 50%–80% eluent B; 3–4 min, 80% eluent B; 4–7 min, 80%–85% eluent B; 7–9 min, 85%–100% eluent B; 9–11 min, 100% eluent B. To quantify the metabolites, a standard curve was established using known levels of each metabolite. The amount of each metabolite in the liver sample was determined based on the standard curve.

### SELECT Method for Detection of m^6^A

To determine the m^6^A methylation status of AML12 cells, the SELECT method was employed.^[^ ^44^ ^]^ In brief, 5 µg of total RNA was mixed with dNTP (5 µmol L^−1^), Up Primer (40 nmol L^−1^), and Down Primer (40 nmol L^−1^) in 1× CutSmart buffer (17 µL, B7204V, NEB, USA). The annealing program consisted of the following steps: 90 °C for 1 min, 80 °C for 1 min, 70 °C for 1 min, 60 °C for 1 min, 50 °C for 1 min, and 40 °C for 6 min. Next, Bst 2.0 DNA polymerase (0.01 U, M0537l, NEB, USA) and SplintR ligase (0.5 U, M0375l, NEB, USA) were added to the mixture. To achieve a final volume of 20 µL, 10 nmol of adenosine triphosphate (P0756S, NEB, USA) was included. The reaction mixture was then incubated at 40 °C for 20 min for the reaction to occur. Subsequently, denaturation was performed at 80 °C for 20 min, and the final reaction mixture was stored at 4 °C. For the qPCR analysis described previously, SELECT assay primers can be found in Table S3 (Supporting Information).

### MeRIP‐qPCR for Detection of m^6^A

To determine the m^6^A concentration of Malat1 lncRNA in AML12 cells, the total RNA was chemically fragmented to an approximate size of 300 nt using a 0.1 mol L^−1^ Tris‐HCl buffer (pH = 7.0) containing 0.1 mol L^−1^ EDTA (pH = 8.0). Following fragmentation, the RNA was purified using the EasyPure RNA Purification Kit (ER701‐01; TransGen, China). Next, the fragmented total RNA (40 µg) was incubated overnight at 4 °C with agarose beads (40 µL, sc‐2003, Santa Cruz, USA). Anti‐m^6^A antibody (68055‐1‐Ig, Proteintech, China) was added to the mixture and incubated overnight at 4 °C. As a negative control, an IgG antibody (2729S, Cell Signaling Technology, USA) was co‐incubated. Immunoprecipitated complexes were captured by overnight incubation with Protein A/G agarose beads (sc‐2003, Santa Cruz, USA). Subsequently, 300 µL of elution buffer (5 mmol L^−1^ Tris‐HCl, pH = 7.4; 1 mmol/L EDTA, pH = 8.0, and 0.05% NaOAc sulfate) along with proteinase K (20 µg) were added to the captured complexes and incubated for 1 h at 4 °C. Input and m^6^A‐enriched RNAs were reverse‐transcribed with random hexamers, and the enrichment was detected by qPCR following phenol extraction and ethanol precipitation. Table S1 (Supporting Information) lists the primers used to detect m^6^A‐enriched transcripts.

### RNA Immunoprecipitation Assay

AML12 cells were cultured in 10 cm dishes and treated with 1% formaldehyde solution for cross‐linking. The cross‐linking reaction was quenched by adding 125 mmol L^−1^ glycine. The cells were then scraped off, collected by centrifugation, and washed with cold PBS buffer. Next, the cells were lysed using IP lysis buffer (EDTA, 1 mmol L^−1^; NaCl, 0.4 mol L^−1^; HEPES, 50 mmol L^−1^, pH 7.5; DTT, 1 mmol L^−1^; glycerol, 10%, and Triton X‐100, 0.5%) containing an RNase inhibitor and 1 mmol L^−1^ PMSF. After sonication, 5 µg of YTHDC1 antibody (ab122340, Abcam, USA) was added and incubated overnight at 4 °C. The mixture was subjected to an overnight incubation with Protein A/G agarose beads to capture the immunoprecipitated complexes. The agarose beads were washed and pelleted by centrifugation. The pellet was resuspended in RIP buffer (NaCl, 0.1 mol L^−1^; HEPES, 50 mmol L^−1^, pH 7.5; DTT, 10 mmol L^−1^; EDTA, 5 mmol L^−1^; 1% SDS; glycerol, 10%, and Triton X‐100, 0.5%) containing an RNase inhibitor and incubated at 70 °C to reverse the cross‐links. The supernatant obtained after centrifugation was employed for RNA extraction, which was performed using the EasyPure RNA Purification Kit (ER701‐01, transGen, China) along with DNase I treatment (2270A, Takara, Japan). The primers listed in Table S1 (Supporting Information) were used for one‐step RT‐qPCR (MF011, Mei5bio, China), and the results were presented as fold enrichment normalized to Input.

### In Vitro Transcription and RNA Pulldown Assay

The pRNA‐SP6‐T7 vector (D2314, Beyotime, China) was utilized for cloning the full‐length mouse Malat1 (NR_0 02847). The vector was linearized and a 5′ overhang was generated at the 3′ end using XhoI. Subsequently, the linearized vector was gel purified and used as a template for in vitro transcription mediated by T7 RNA polymerase (TR101, Vazyme, China). To generate biotinylated RNA probes complementary to Malat1 lncRNA, streptavidin magnetic beads (P2151, Beyotime, China) were incubated with biotinylated RNA probes at the 5′ end for 1 h at room temperature. The synthesized RNA was isolated using the EasyPure RNA Purification Kit (ER701‐01, TransGen, China) with DNase I treatment (2270A, Takara, Japan) to remove template DNA. AML12 cells were treated with IP lysis buffer (P0013, Beyotime, China), followed by thorough mixing with RNA‐bound beads, and incubated overnight at 4 °C. The samples were then eluted, and western blot analysis was performed to analyze YTHDC1 and NRF2. The sequences of the biotin‐labeled probes were as follows: Malat1, 5′‐TTCGATAAGCTACTCTATTAGC‐3′; Random probe, 5′‐TTCTTACTACGTGAGGTCGT‐3′ (RiboBio, China).

### Library Construction and RNA‐Seq

TRIzol Reagent (TSP401, Tsingke Biotech, China) was utilized to extract total RNA. The purity and quality of the extracted total RNA were assessed using the vendor's recommended protocol, and the purification, library construction, and sequencing were outsourced to Lianchuan Biotechnology Co., Ltd. in Hangzhou, China.

### Statistical Analysis

The data were presented as mean ± standard deviation (SD) and analyzed using SPSS 20.0 (IBM, Armonk, NY). The differences between groups were compared using either student's t‐test or two‐way analysis of variance (ANOVA) followed by Tukey's test for multiple comparisons, and statistical significance was considered at *P* < 0.05.

## Conflict of Interest

The authors declare no conflict of interest.

## Author Contributions

Y.F. and J.S. contributed equally to this work. Y.F. and J.S. prepared and executed the experiments; Y.F. analyzed the data and wrote the manuscript; J.S. Z.L, Z.C., and M.Z. assisted with experiments and provided technical assistance. X.M. designed the experiments and resourced the projects. All authors have read and approved the final version of the manuscript.

## Supporting information

Supporting Information

## Data Availability

The data that support the findings of this study are available from [PARTY]. Restrictions apply to the availability of these data, which were used under license for this study. Data are available at PRJNA887853 with the permission of [PARTY].
