# Supplementary material for: PXR Activation Relieves Deoxynivalenol‐Induced Liver Oxidative Stress Via Malat1 LncRNA m6A Demethylation
Source: Adv Sci (Weinh). 2024 Apr 24;11(25):2308742. doi: 10.1002/advs.202308742 (PMC11220637; doi:10.1002/advs.202308742)
Supplement: Supplementary file 1 — Supporting Information [file ADVS-11-2308742-s001.pdf]

ADVANCED SCIENCE
Open Access

Supporting Information

for *Adv. Sci.*, DOI 10.1002/advs.202308742

PXR Activation Relieves Deoxynivalenol-Induced Liver Oxidative Stress Via Malat1 LncRNA m^6A Demethylation

*Yue Feng, Jiakun Shen, Zishen Lin, Zeyi Chen, Min Zhou and Xi Ma**

Supporting Information

PXR Activation Relieves Deoxynivalenol-Induced Liver Oxidative Stress Via Malat1 LncRNA m^6A Demethylation

Yue Feng[‡], Jiakun Shen[‡], Zishen Lin, Zeyi Chen, Min Zhou, and Xi Ma[*]

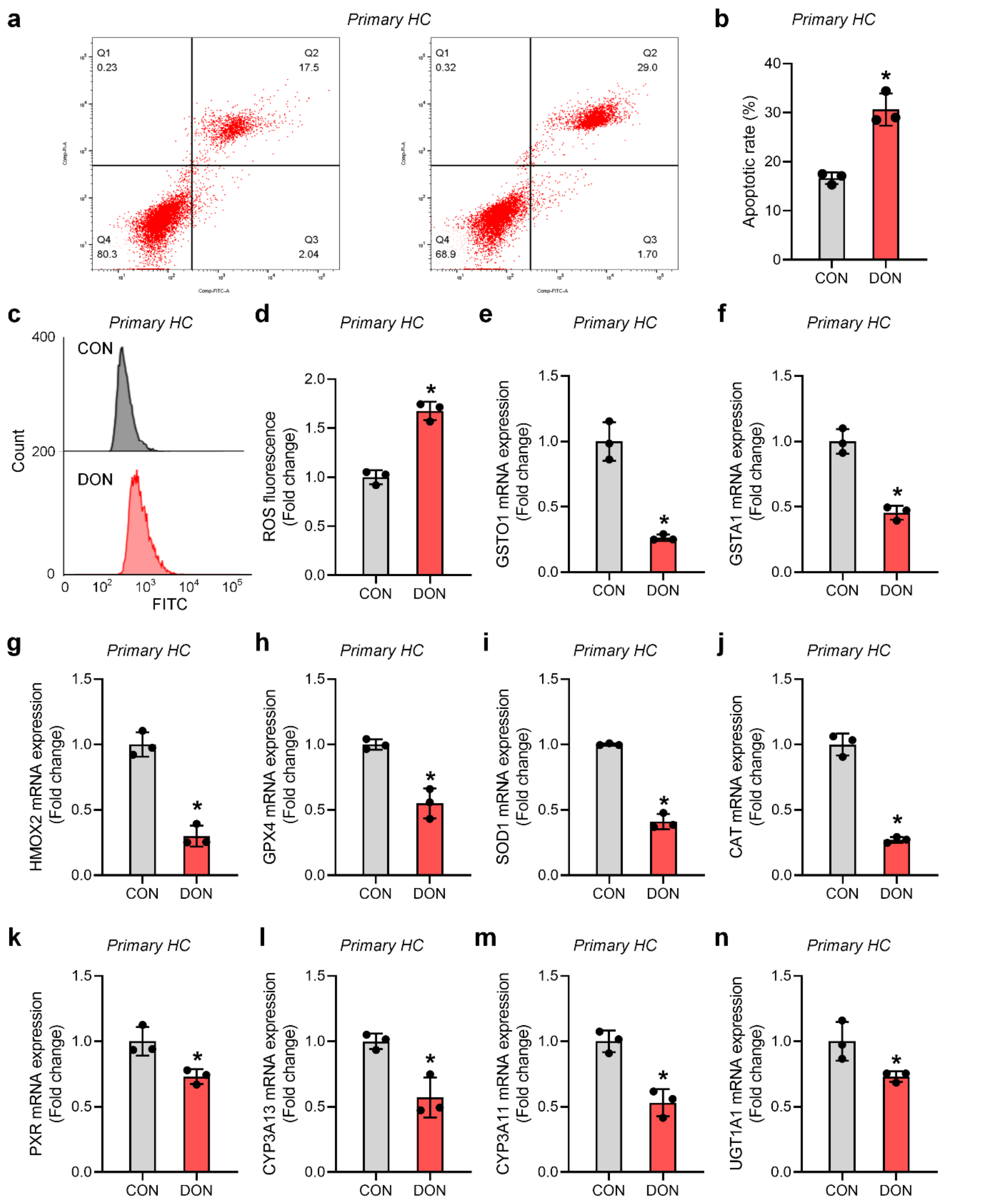

Figure S1. DON treatment induced oxidative stress and apoptosis in primary hepatocytes, accompanied by transcriptional repression of PXR and downstream transcripts. a, b) The effect of DON treatment on the apoptosis rate of primary hepatocytes (n = 3). c, d) The effect of DON treatment on the ROS content of primary hepatocytes (n = 3). e-j) The effect of DON treatment on the mRNA levels of antioxidant-related factors in primary hepatocytes (n = 3). k-n) The effect of

DON treatment on the mRNA levels of PXR and its downstream transcripts in primary hepatocytes (n = 3). Values are expressed as mean ± standard deviation, *$P < 0.05$.

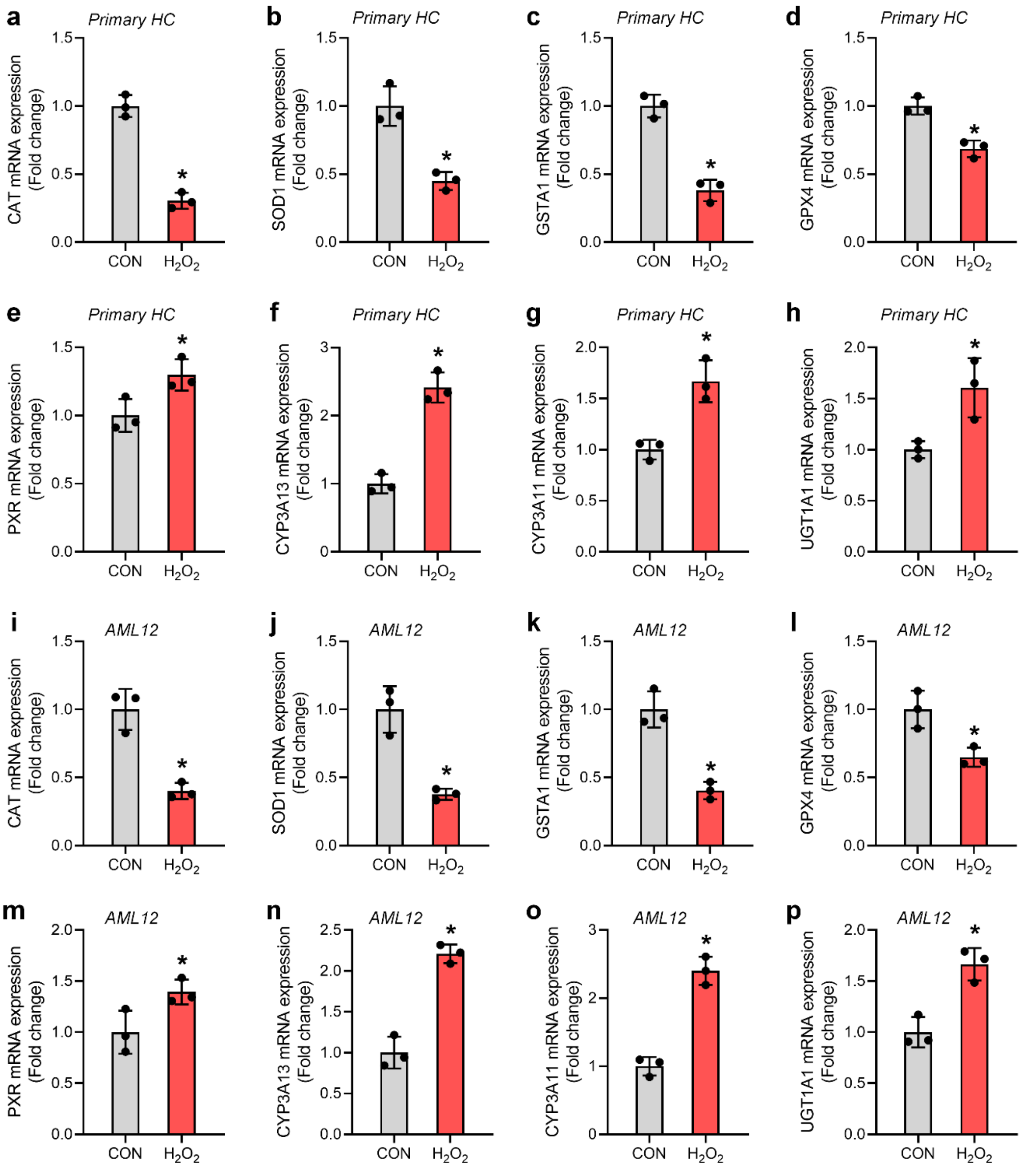

Figure S2. Expression patterns of PXR and its downstream transcripts in the oxidative stress model induced by H_2O_2 treatment. a-d) The impact of H_2O_2 treatment on oxidative stress-related indicators in primary hepatocytes (n = 3). e-h) The effects of H_2O_2 treatment on mRNA levels of PXR and its downstream transcripts in primary hepatocytes (n = 3). i-l) The influence of H_2O_2 treatment on oxidative stress-related indicators in primary hepatocytes (n = 3). m-p) Effects of H_2O_2 treatment on mRNA levels of PXR and its downstream transcripts in primary

hepatocytes (n = 3). Values are expressed as mean ± standard deviation, *P < 0.05.

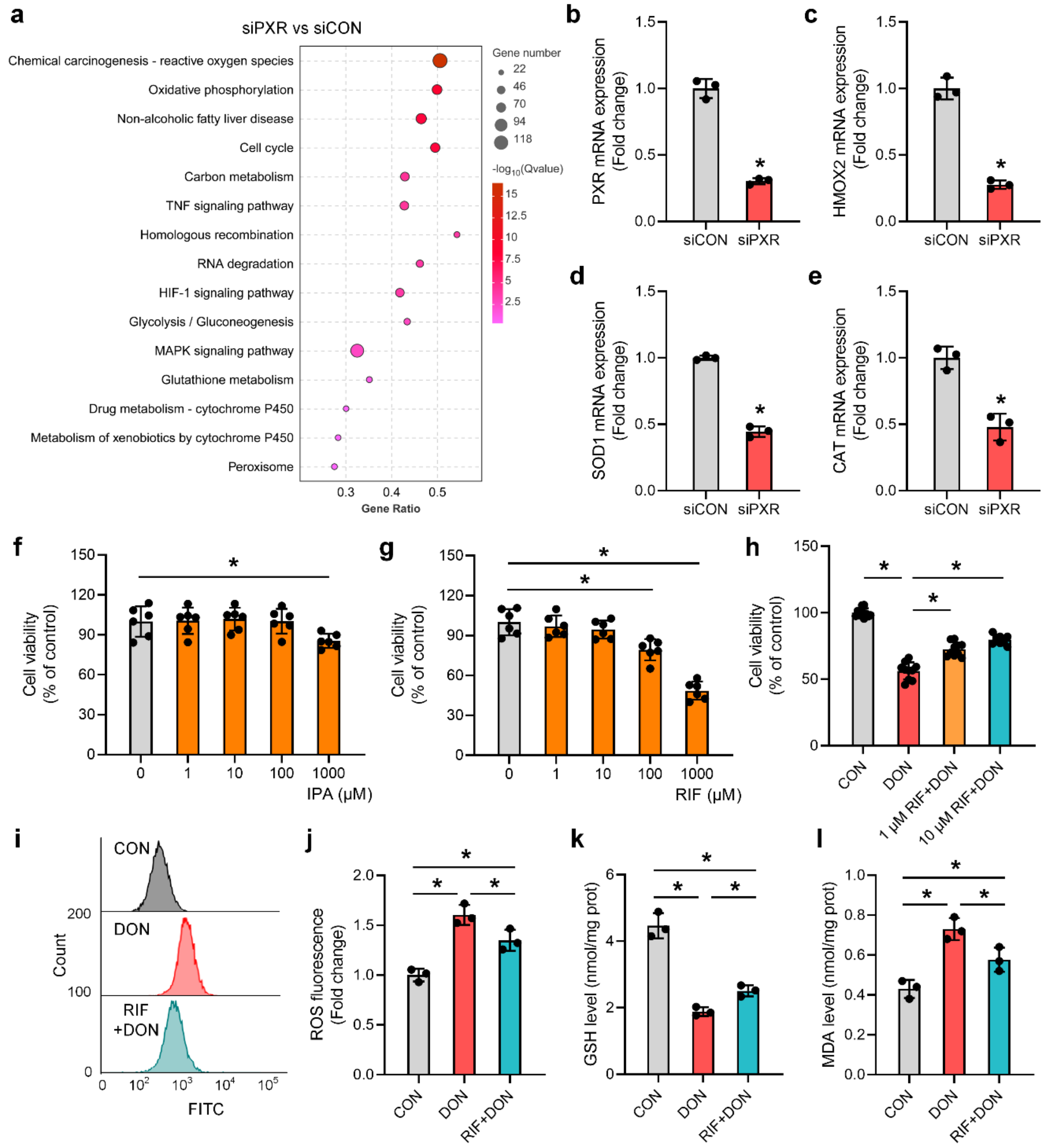

Figure S3. PXR regulates the oxidative stress process in hepatocytes. a) Knockdown of PXR in hepatocytes resulted in significant enrichment of oxidative stress-related pathways, including chemical carcinogenesis-reactive oxygen species, oxidative phosphorylation, and glutathione metabolism, as revealed by RNA-seq results (n = 3). b) Validation of PXR knockdown efficiency at the mRNA level by PXR siRNA transfection in hepatocytes (n = 3). c-e) Effects of PXR knockdown on mRNA levels of oxidative stress-related factors in hepatocytes (n = 3). f, g) Identification of the optimal concentrations of IPA and RIF for treating AML12 cells (n = 6). h) The protective effect of the PXR agonist RIF on DON-induced

hepatocyte activity decline (n = 8). i, j) The mitigating effect of the PXR agonist RIF on DON-induced hepatocyte ROS accumulation (n = 3). k, l) The protective effect of the PXR agonist RIF on the disruption of oxidative stress-related key enzymes MDA and GSH levels induced by DON in hepatocytes (n = 3). Values are expressed as mean ± standard deviation, *P < 0.05.

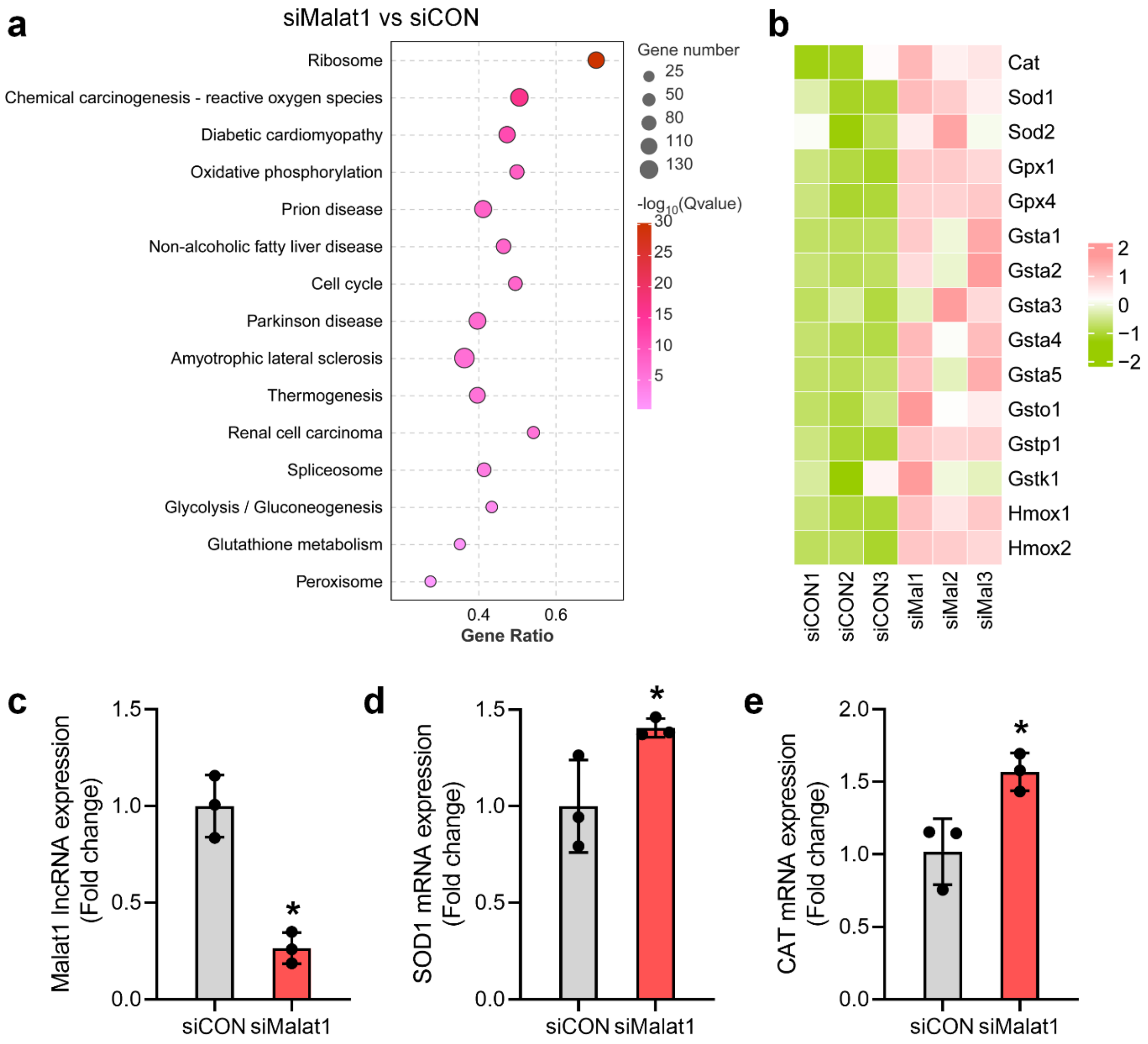

Figure S4. Malat1 lncRNA regulates the oxidative stress process in hepatocytes. a) Knockdown of Malat1 in hepatocytes resulted in significant enrichment of oxidative stress-related pathways, including chemical carcinogenesis-reactive oxygen species, oxidative phosphorylation, and glutathione metabolism, as revealed by RNA-seq results (n = 3). b) Heatmap analysis showed the effect of Malat1 knockout on the transcriptional expression levels of antioxidant-related factors in hepatocytes (n = 3). c) Validation of Malat1 lncRNA knockdown efficiency was achieved through Malat1 siRNA transfection in hepatocytes (n = 3). d, e) Knockdown of Malat1 in hepatocytes resulted in alterations in mRNA levels of oxidative stress-related factors (n = 3). Values are expressed as mean ± standard deviation, *P

< 0.05.

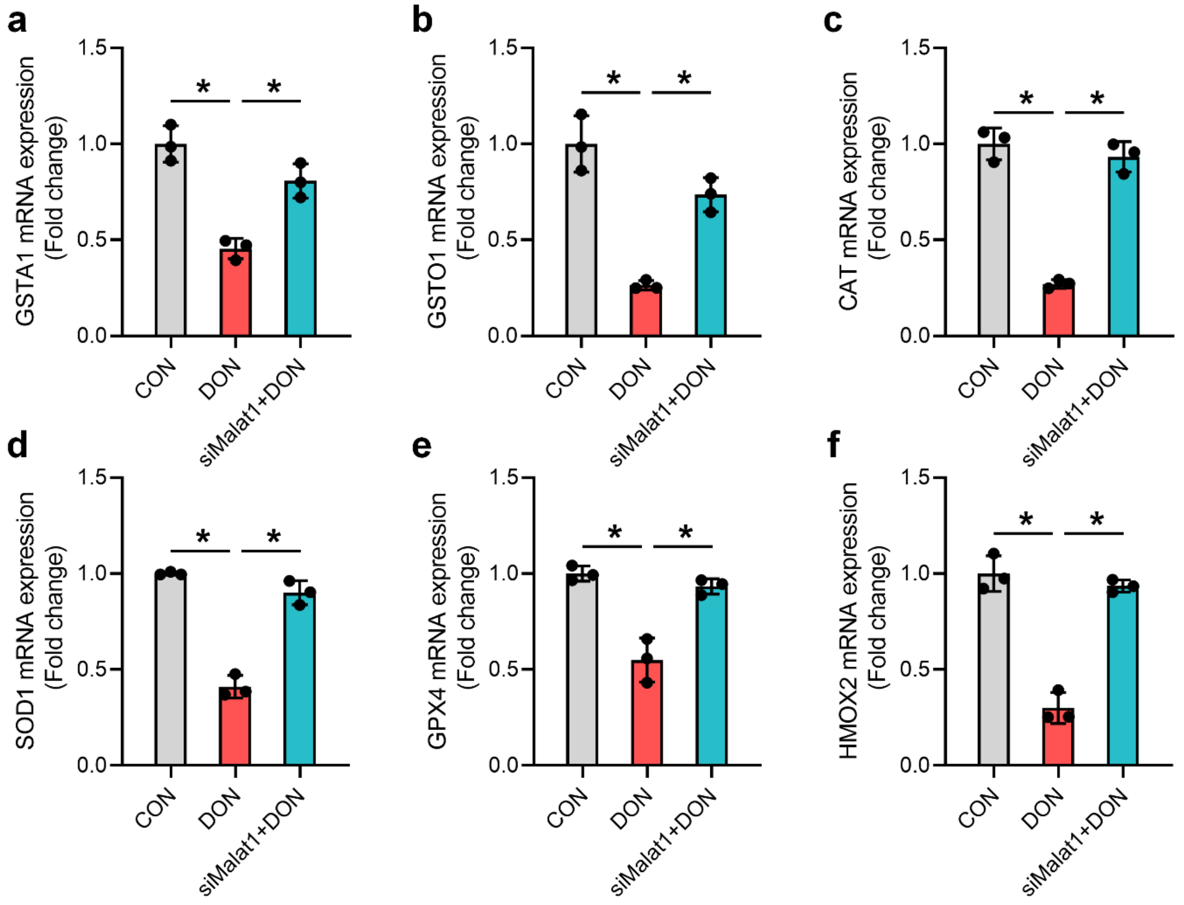

Figure S5. Malat1 lncRNA mediates the impairment of antioxidant function in hepatocytes induced by DON. a-f) Knockdown of Malat1 alleviated the reduction of mRNA levels of antioxidant-related factors induced by DON treatment in AML12 cells (n = 3). Values are expressed as mean ± standard deviation, *P < 0.05.

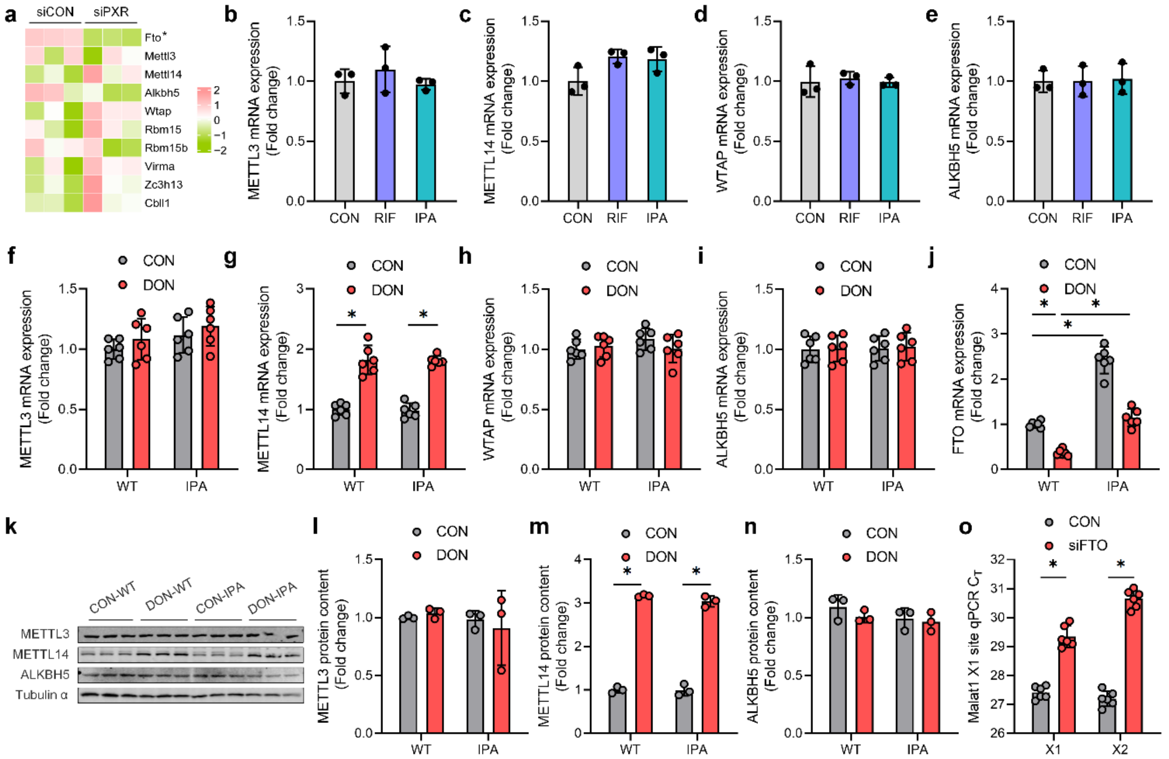

Figure S6. Regulation of PXR on the expression levels of m^{6}A modification-related enzymes in hepatocytes. a) Heatmap analysis showed the regulatory effect of PXR knockdown on the transcriptional levels of m^{6}A modification-related enzymes (n = 3). b-e) Effects of PXR agonists RIF and IPA treatment on the mRNA levels of m^{6}A methyltransferases METTL3/14, WTAP, and demethylase ALKBH5 (n = 3). f-j) Effects of IPA and DON treatment, alone or in combination, on the mRNA levels of m^{6}A modification-related enzymes (n = 6). k-n) Effects of IPA and DON treatment, alone or in combination, on the protein levels of m^{6}A modification-related enzymes (n = 3). o) The SELECT method was used to detect m^{6}A modifications in the Malat1 lncRNA m^{6}A modification site (n = 6). Values are expressed as mean ± standard deviation, *$P < 0.05$.

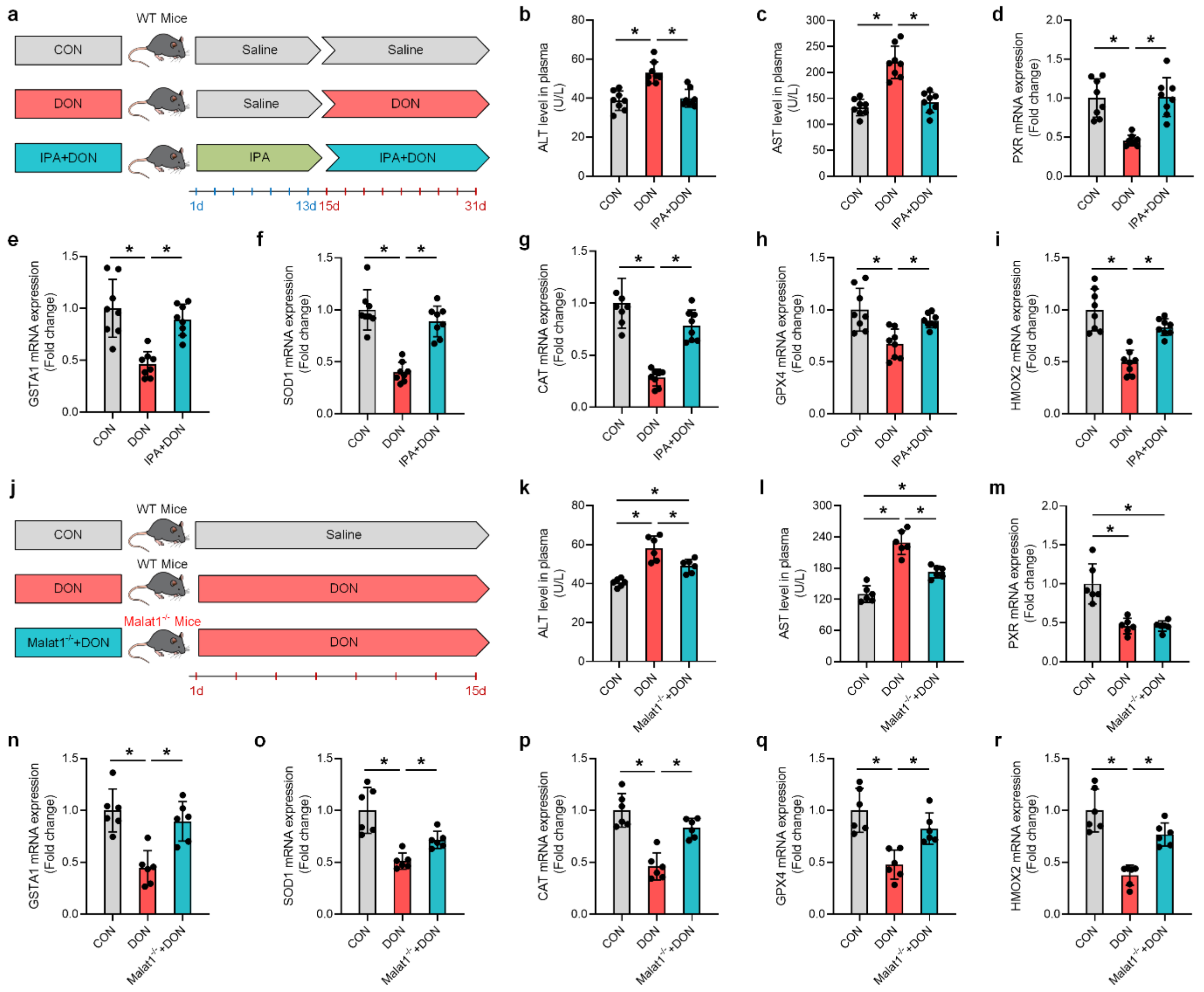

Figure S7. IPA and Malat1 knockout alleviated DON-induced oxidative stress and liver injury in mice. a) Experimental design schematic for IPA and DON gavage. b, c) IPA treatment reduced the levels of liver injury markers AST and ALT in the livers of DON-treated mice (n = 8). d) Effects of IPA and DON treatment on the expression level of PXR mRNA in the liver (n = 8). e-i) Effects of IPA and DON treatment on mRNA expression levels of antioxidant-related factors in the liver (n = 8). j) Experimental design schematic for DON gavage in WT mice and Malat1$^{-/-}$ mice. k, l) Knockout of Malat1 reduced levels of liver injury markers AST and ALT induced by DON (n = 8). m) Effects of Malat1 knockout and DON treatment on the expression level of PXR mRNA in the liver (n = 8). n-r) Effects of Malat1 knockout and DON treatFment on mRNA expression levels of antioxidant-related factors in the liver (n = 8). Values are expressed as mean ± standard deviation, *$P < 0.05$.

Table S1. Nucleotide sequences of primers.

Target genes	Primer sequences (5'to 3')	Used for
PXR	F: AGAGATCATCCCTCTTCTGCCAC<br>R: GATCTGGTCCTCAATAGGCAGGT	Real-time PCR
METTL3	F: CAGTGCTACAGGATGACGGCTT<br>R: CCGTCCTAATGATGCGCTGCAG	Real-time PCR
METTL14	F: AGAGTGCGGATAGCATTGGTGC<br>R: CTCCTTCATCCAGACACTTCCG	Real-time PCR
FTO	F: GCCTCGGTTTAGTTCCACTCAC<br>R: GTCGCCATCGTCTGAGTCATTG	Real-time PCR
WTAP	F: GAACCTCTTCCTAAAAAGGTCCG<br>R: TTAACTCATCCCGTGCCATAAC	Real-time PCR
ALKBH5	F: ACCACCAAGCGGAAATACCAG<br>R: TGGTAGTCGTTGATGACCGC	Real-time PCR
Malat1	F: AATGTTAAGAGAAGCCCAGGG<br>R: AAGGTCAAGAGAAGTGTCAGC	Real-time PCR
GSTO1	F: CGAACCTAAGGGAAGCGTTGGA<br>R: TTCCAGTCGCTGAAACCAAGGC	Real-time PCR
GSTA1	F: CTGCCTTGGCAAAAGATAGGACC<br>R: CTTCCAGTAGGTGGATGTCCAC	Real-time PCR
CAT	F: GGTGCCCCCAACTATTACCC<br>R: TCCGCACCTGAGTGACATTG	Real-time PCR
SOD1	F: GGTGAACCAGTTGTGTTGTCAGG<br>R: ATGAGGTCCTGCACTGGTACAG	Real-time PCR
GPX4	F: CCTCTGCTGCAAGAGCCTCCC<br>R: CTTATCCAGGCAGACCATGTGC	Real-time PCR
HMOX2	F: CAAGGAAGCACATGACCGAGCA<br>R: CTTGTTGCGGTCCATTTCCTCC	Real-time PCR
UGT1A1	F: TTGTGTGTGTTCGGTCCCT<br>R: CAGTCCGTCCAAGTTCCACC	Real-time PCR

PGLYRP2	F: CCTTCGTGGGCAACTACACT<br>R: CCTCTGTGAAGTGAGGCCAG	Real-time PCR
CYP3A11	F: TCAGCTCTCTCACTGGAAACC<br>R: TTAATCGTCTCTGGGTCTGTGAC	Real-time PCR
CYP3A13	F: AACCTCTGCCTTTCTTGGGG<br>R: ATACCCACTGGACCAAAGCG	Real-time PCR
PPIA	F: CATACAGGTCCTGGCATCTTGTC<br>R: AGACCACATGCTTGCCATCCAG	Real-time PCR
FTO	F: CTTTTTCAAGGGAAGCCCAGG<br>R: AAGCAATAAGCCACAGCAGG	ChIP-PCR
Malat1 1-361 nt	F: CAGGCATTCAGGCAGCGA<br>R: CCAGCCGGAACATCTAA	MeRIP-qPCR
Malat1 292-742 nt	F: TAGGAGATTGTAAAGGGA<br>R: CTCTTTCTTCTAATCTTTTA	MeRIP-qPCR
Malat1 635-1104 nt	F: GCCTGGTCTACAGAGTG<br>R: CTTCGATCGTTAGTTTCG	MeRIP-qPCR
Malat1 1039-1435 nt	F: ATGGAGCGAGCAGTTTAG<br>R: CTTCTATCGGGTCCTTTC	MeRIP-qPCR
Malat1	F: ATGGAGCGAGCAGTTTAG<br>R: CTTTCCTGGGCTATCTTC	RIP-qPCR

Table S2. Nucleotide sequences of siRNA.

Target	Target sequance
Malat1	GCAGUUUAGGAGAUUGUAAAG
YTHDC1	GUAUGAUGGUUUGACUAUAUG
PXR	GCAGUUGCUGCGCAUCCAA
FTO	GUCUCGUUGAAAUCCUUUGAU
PGLYRP2	GCUGCCACUUGGAUUCUUAUA
Negative control	siN0000001-1-5, RiboBio

Table S3. Nucleotide sequences of SELECT method.

Target	Sequences (5'to 3')
Malat1 lncRNA X1 site	Up Probe: tagccagtaccgtagtgcgtgCTTGAAACTGTTATCAAAAG<br>Down Probe: CCTTTTGTCATCAAGCAAAAcagaggctgagtcgctgcat
Malat1 lncRNA X2 site	Up Probe: tagccagtaccgtagtgcgtgTGCTGCTGCTGGCTCCTCAG<br>Down Probe: CCTTCCTAGCTTCACCAAACcagaggctgagtcgctgcat
Malat1 lncRNA N site	Up Probe: tagccagtaccgtagtgcgtgTTTTAATATTCTTCTAACTC<br>Down Probe: TCAAAGGCATTCTGCCTTAAcagaggctgagtcgctgcat
qPCR	Forward Prime: ATGCAGCGACTCAGCCTCTG<br>Reverse Prime: TAGCCAGTACCGTAGTGCGTG

Name	Score	Start	End	Strand	Predicted sequence
MA1533.1.NR1I2	10.8712	758	774	+	ATGCCCTGAGTAGACTC
MA1533.2.NR1I2	9.4412	759	773	+	TGCCCTGAGTAGACT

Table S4. Nucleotide sequences of FTO promoter and PXR binding sites.

>FTO promoter (5' Flanking sequence) chromosome: GRCm39: 8:92038153:92040152:1

GTGCATGAAGAGTCTCTCTTAGAGAGAGGGCCTGGAACTTTGATTGGAACTCATGATGGGCTGAGA
ACCACTGCTAGGGGCAAATATCTGAAAGAATGTCTGCAGAAGTTGATGCTAACACATATTGTTCACT
GTGCTTACAGTGTGCGTAAGCAATGGCTACTCAAATGGTTCCTGAGATACAAACAGAAGCTAAGAC
AAGCTAGCTCAATTGCTCAAGGTCATTAGATACAAACAGAAGCTAAGACAAGCTAGCTCAATTGCTC
AAGGTCATTAATGCAGGAGTTAGTTGTTTGGTGATATTTCTGGTTATCATGACCGAGAAGAAAATTGTT
ACTGGTCTGATGTAGGTAGATGGCACAGTGAATTCTCCCTCTTTGCTATGATCCACTTACAACCTTC
CCTGCTTTCCTCACATATGGCATGCTTTTATCTTGCTCCTAAGGCTCTTCTGTCTCCTCATACTTCAGA
GCTCAGTCCAACTGACCTTTCCTTAGAGTTTCCCTTACTGTTCTGTGTTAATTTTCTTCCTGGTTTCTAA
CTTTTCTTTTTCAAGGGAAGCCCAGGCTGTCTAGGCTGACATGAAATTCACCAAGAACACTAAACAT
ACTGGCCTCAAATTCTCTATTTTTTCCTTGTGTTTTTTTTTTCTTTAAAAAATGGGGGGGGGGTCATCT
CACTATATAAACTTGGCTGGAATTCAAAGATCTCCCTGCCTCTACTTCCAGAGTGTTACGATTAAAGT
CGGGTGTCACC**ATGCCCTGAGTAGACTC**TGAATTTGTCCTGAGTGTGCCACCACCCTGCTGTGGC
TTATTGCTTACAGCAACAGCCTTTGTCATTATCCCAGAGTTGTATCGTAATTCATTGTCATGTCATGCA
TTTTTAACCTAGTATATTTTCACACCTATATCATAGTTTCTTGCTTATATCTGTCCCCTACCACTCCCCC
CCCCATCTCTCTAGCTTCCTCTCACTGGTCTCCTTCCTCCCCCAAATAGTTCCTCTTCTGCTATCTG
ATGTCACATAGTAGACATTAACGCTATCATTTATTGAATGAATGAATGAATGAATGAATGCCAGGCAC
TGAGTTTAAACAAAGTCTCTGCCCTGTAATGGCTTTAACAGACTATGGGAGTAGTCAGATGAGAAAC
AGTAATAAAGAATAAAAGATAGGCCACTAGAAACTGATTGTGGTGGTGGGAACTTGAGGCAAGACCT
ATTATTGATTGAATTTGAGACGAGCATGGGCTAAAAAGGCTAGTTTAAGGCCAGCCTAGGATGCATA
GGGAGACCCCCTTTTAAAAACTAATCGACAATCAGCAGCAGCAGCAACAACAACAACAGTAACAA
GCCAACAACAACAACAGTAACAAGCCTCTTGGGAGGCTTAGGTTCTACCATCCAAGCCCAGTTTCT
GGGAAACAGAGTGAAATGTGACTGGGACCTTTGAGTTGTAGGACTTAGGGTTGCCAATTCTTATCAG
CTGCGTTTCTGGCTGGGCGGGCGGTCAGGGCGAGTCAGATGCAGGCCTGCGGATGTGGAGGTG
TGTCGGGATGGACTGCTTTCCACGCAAGAATCCCAGGTCTATCCTGGGCTTCGAACAGACTCCAG
GCTCAGTTGTGAGGCCGCGGAAGAGCCAGCCAACCTGCATTAGCCCCCACCTCACAGAGTACTC
ACCTCCGCGTTCGCCCTCAGCTAGCTACCGGTTGCTATAGCGCCCAGGGCGCGAGCCTCTGCA
GGCCCAGAGGAGCACGGGAAAACAGTGAGTCCCGTTACCCACTGGGAAATGTAGTTCTCCGTAG
GGTGCGGGCCGTCGGCGGGTCGCGTAGCGGACTACGCTAGCCCTGCTAGCTGACTGGAGAAAT
TCAGCTCCGGAGCCCAAGGAGTACTCGGGAGTCTCGCGCAGCATTCTGGGAAGGGTAGTTCTTCT
AGGTATTAGAGCAAATGGCTCCAGGCGGGAGAGAGCCGTTGAGAGAACTACACACAGGAGGCGG
GGTCC
